# DNA supercoiling in bacteria: state of play and challenges from a viewpoint of physics based modeling

**DOI:** 10.3389/fmicb.2023.1192831

**Published:** 2023-10-30

**Authors:** Ivan Junier, Elham Ghobadpour, Olivier Espeli, Ralf Everaers

**Affiliations:** ^1^CNRS, UMR 5525, VetAgro Sup, Grenoble INP, TIMC, Université Grenoble Alpes, Grenoble, France; ^2^École Normale Supérieure (ENS) de Lyon, CNRS, Laboratoire de Physique and Centre Blaise Pascal de l'ENS de Lyon, Lyon, France; ^3^Center for Interdisciplinary Research in Biology (CIRB), Collège de France, CNRS, INSERM, Université PSL, Paris, France

**Keywords:** DNA supercoiling, bacterial DNA, physical modeling, DNA replication, gene transcription, nucleoid, multi-scale modeling

## Abstract

DNA supercoiling is central to many fundamental processes of living organisms. Its average level along the chromosome and over time reflects the dynamic equilibrium of opposite activities of topoisomerases, which are required to relax mechanical stresses that are inevitably produced during DNA replication and gene transcription. Supercoiling affects all scales of the spatio-temporal organization of bacterial DNA, from the base pair to the large scale chromosome conformation. Highlighted *in vitro* and *in vivo* in the 1960s and 1970s, respectively, the first physical models were proposed concomitantly in order to predict the deformation properties of the double helix. About fifteen years later, polymer physics models demonstrated on larger scales the plectonemic nature and the tree-like organization of supercoiled DNA. Since then, many works have tried to establish a better understanding of the multiple structuring and physiological properties of bacterial DNA in thermodynamic equilibrium and far from equilibrium. The purpose of this essay is to address upcoming challenges by thoroughly exploring the relevance, predictive capacity, and limitations of current physical models, with a specific focus on structural properties beyond the scale of the double helix. We discuss more particularly the problem of DNA conformations, the interplay between DNA supercoiling with gene transcription and DNA replication, its role on nucleoid formation and, finally, the problem of scaling up models. Our primary objective is to foster increased collaboration between physicists and biologists. To achieve this, we have reduced the respective jargon to a minimum and we provide some explanatory background material for the two communities.

## 1. Introduction

With respect to DNA, efficient growth and division of bacteria rely on two major processes: (i) an appropriate expression of the genetic program allowing the generation in the right amounts and proportions of the proteins and enzymes necessary for the duplication of cells; (ii) a faithful replication of DNA and a reliable segregation of the replicated chromosomes during cell division. Research over the last fifty years or so has shown that the analysis of the topological constraints inherent in the double-helix nature of DNA is crucial for a quantitative understanding of these problems (Wang et al., 1983; Travers and Muskhelishvili, 2005; Dorman and Dorman, 2016). Topological constraints are more particularly responsible for the supercoiling of bacterial DNA, i.e., the under or overwinding of bacterial DNA, which is known to impact all levels of chromosome structure (Wang et al., 1983; Travers and Muskhelishvili, 2005; Badrinarayanan et al., 2015; Dorman and Dorman, 2016; Dame et al., 2020; Lioy et al., 2021a).

Just as most fields of biology, investigation in the field of DNA supercoiling has recently thrived thanks to a dramatic acceleration in the production of experimental results as a result of low-cost DNA sequencing, new genome engineering techniques and the development of visualization methods of increasing resolution. One of the consequences of having access to comprehensive data, some of which, such as high-throughput chromosome conformation capture (Hi-C) data (Lieberman-Aiden et al., 2009), covers almost all scales of a chromosome (Lieberman-Aiden et al., 2009; Le and Laub, 2014), is the possibility of building models of chromosomal organization across multiple genomic scales. In this regard, it is essential to consider that the term *model* can have different meanings depending on the scientists' background, including biologists, modelers, and those with or without a physical background. For instance, in the context of chromosome structuring, *data-driven models* (Rosa and Zimmer, 2014; Imakaev et al., 2015; Junier et al., 2015) involve many parameters that may not be associated with any physical mechanism but, instead, used to generate, within a given polymer framework, chromosome conformations that are compatible with genome-wide data (Umbarger et al., 2011; Zhang and Wolynes, 2015; Messelink et al., 2021) – generated conformations can then be used to explore the statistical properties that underlie experimental data (Zhang and Wolynes, 2015; Messelink et al., 2021). On the other hand, *physics-based models* involve a set of physically motivated parameters, often parsimonious, and are used to *rationalize* observed experimental data within the framework of the fundamental laws of Physics, particularly within the realm of Statistical Mechanics. In the case of DNA, the employed models often come from the neighboring fields of polymer physics and of soft and active matter (Marko, 2015).

In this review, we aim to discuss the problem of DNA supercoiling from this perspective of physical modeling, examining the components of biophysical models, their outcomes, as well as their limitations and possible workarounds. By doing so, we aim to clarify the open problems in the field, following the line of the famous quote by Richard Feynman: “What I cannot create, I do not understand.” To this end, we have divided the review into seven sections plus an Appendix (Supplementary File). Section 2 revisits essential notions of DNA topology, introduces the molecular machines central to the problem, and discusses the problem of *in vivo* measurements of DNA supercoiling. In Section 3, we introduce the modeling approaches employed by biophysicists to comprehend and predict the behavior of supercoiled DNA, with additional details provided in the Appendix. Section 4 presents the main steps marking the development of models aiming at capturing the *equilibrium properties* of supercoiled DNA, along with a discussion of their relevance for *in vivo* situations. Sections 5, 6 focus on transcription and replication, respectively, emphasizing the necessity to build *far from equilibrium models* that involve not only the transcription and replication machineries but also the action of topoisomerases. In Section 7, we discuss the formation of the nucleoid, which is the membrane-free region of the bacterial cells where DNA is found. In the final Section 8, we review the attempts to model the structuring of bacterial chromosomes at the largest scales.

## 2. DNA supercoiling in bacteria: fundamentals

DNA is a polymer made up of nucleotides, arranged in a double helix structure formed by two intertwined strands, known as Watson and Crick strands, which are held together by hydrogen bonds. In its relaxed state, at typical physiological temperature and salt concentration, a DNA double helix contains approximately 10.5 base pairs (B-DNA form). However, in mesophilic bacteria, i.e., in bacteria living under mild conditions of temperature, pressure and pH, the double helix is generally longer, containing more than 10.5 base pairs. Bacterial DNA is therefore under torsional stress, with an average underwound or, equivalently, negatively supercoiled double helix. This section explores the reasons behind these observations, starting with the notion of the linking number, the role of topoisomerases in relaxing torsional stresses generated during gene transcription and DNA replication, and the challenges of measuring the supercoiling properties of bacterial chromosomes.

### 2.1. Linking number, twist/writhe decomposition and structural consequences

**Figure 1:**
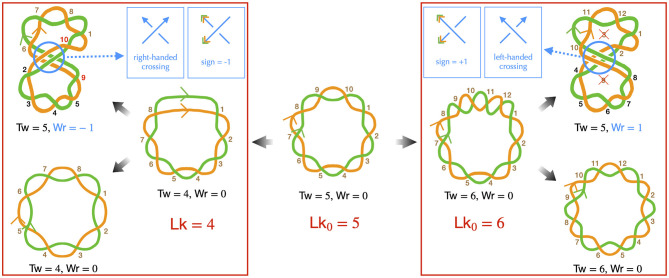
Implications of the linking number conservation in circular DNA —only the DNA strands are depicted (in green and orange), presented schematically to emphasize topological properties. **Center**: we consider a reference molecule, torsionally relaxed and planar (Wr = 0), consisting of five helix turns (Tw = 5), resulting in a relaxed linking number Lk_0_ = 5. Brown numbers indicate right-handed DNA helix crossings. **Left**: we remove one helix turn (negative supercoiling), resulting in a molecule with heterogeneous helicity, comprised of four helix turns (right conformation), leading to Lk = 4 with Tw = 4 and Wr = 0. Note that the half-turn at the top of this conformation strongly distorts the double helix and likely denatures in real situations. One possibility is that the helix turns redistribute, achieving homogeneous helicity (bottom left conformation), the writhe and twist remaining unchanged. Alternatively, the molecule may buckle, forming a super-structure (top left conformation). In this case, the molecule can recover its relaxed twist (Tw = 5) if the super-structure is right-handed, with a corresponding Wr = −1, by allowing the strands to cross two more times around the main axis, as indicated by the red numbers. In this conformation, the black numbers indicate helix crossings with a change in the strand passing on top of the other one, as a consequence of the buckling, the handedness of the helix remaining unchanged. **Right**: we introduce one helix turn (positive supercoiling). Qualitatively, the discussion resembles that of negative supercoiling, with one notable difference: to achieve the relaxed twist, a helix turn must be removed, not added. As indicated by the crossed numbers, this can occur with a left-handed super-structure, characterized by Wr = +1. Finally, we remind that determining the handedness of the super-structure is based on the same rule as for the DNA double helix to indicate the directions of the main axis (blue arrows in the top inset panels). The sign of the corresponding writhe is instead determined using the directions as given by the DNA strands (orange and blue arrowheads in the top inset panels).

The DNA of most bacteria exists in a circular form. This characteristic has specific implications at all levels of bacterial chromosome structuring, ranging from the base pair to the large-scale chromosome conformation. The various conformations the chromosome can adopt must indeed be consistent with the so-called *conservation of the linking number*. Specifically, the linking number (Lk) of a circular DNA molecule represents the number of times the two DNA strands intersect in the three-dimensional space. For example, in a planar molecule, Lk is equal to the number of helix turns the two strands make along the molecule's central axis—this can be calculated by considering that one helix turn of B-DNA consists of approximately 10.5 base pairs. In the more general case of a three-dimensional molecule, the strand intersections can occur locally as the strands twist around each other along the molecule's central axis, as well as globally when the main axis folds and crosses itself (Figure 1). Consequently, Lk is the combined result of the twist (Tw) and the writhe (Wr), expressed as Lk = Tw+Wr (Calugareanu, 1959; White, 1969; Fuller, 1978). The twist refers to the total number of helix turns, while the writhe represents the average number of times the main axis crosses itself from any perspective (Fuller, 1978).

*DNA supercoiling* occurs when the linking number deviates from that of the corresponding mechanically relaxed molecule. This can happen *in vivo* due to the activity of enzymes, as discussed below, or *in vitro* when the DNA is manipulated, for example, by magnetic tweezers (Strick et al., 2003). Note that, conventionally, a positive contribution to the twist indicates a helix involving right-handed intersections, while a positive contribution to the writhe signifies a left-handed intersection in space. Conversely, negative contributions for twist and writhe indicate a left-handed double helix and a right-handed intersection, respectively (Figure 1).

Importantly, for any deformation of the DNA molecule in which the two strands are not cut, the linking number remains unchanged (Calugareanu, 1959; White, 1969). This property, known as the *conservation of the linking number*, implies that twist can precisely convert into writhe, and *vice versa*, as depicted in Figure 1—describing DNA as a ribbon can further help to apprehend this property (Crick, 1976; Bauer et al., 1980). This fundamental characteristic enables supercoiled DNA to relieve local torsional stress by generating super-structures, such as plectonemes. The relative proportions of deformations in the double helix and formation of super-structures are then determined by the energy costs associated with torsion and bending mechanical properties of DNA. Physical models have extensively focused on predicting both these proportions and the resulting conformations, as explained in detail in Sections 3, 4.

### 2.2. Topoisomerases: changing the DNA linking number when resolving transient topological stresses

When a circular B-DNA molecule is in its mechanically most relaxed state, the twist is close to the number of double helix turns, the writhe is negligible with respect to the twist, and the corresponding relaxed linking number, Lk_0_, is almost equal to the twist. However, *in vivo*, DNA undergoes torsional stresses generated during DNA replication and gene transcription. These stresses are alleviated by DNA enzymes called topoisomerases (Wang, 2002; Forterre et al., 2007; McKie et al., 2021). By doing so, topoisomerases effectively change the overall linking number of DNA, leading to supercoiling of the bacterial chromosome wherein the linking number differs from Lk_0_.

**Figure 2:**
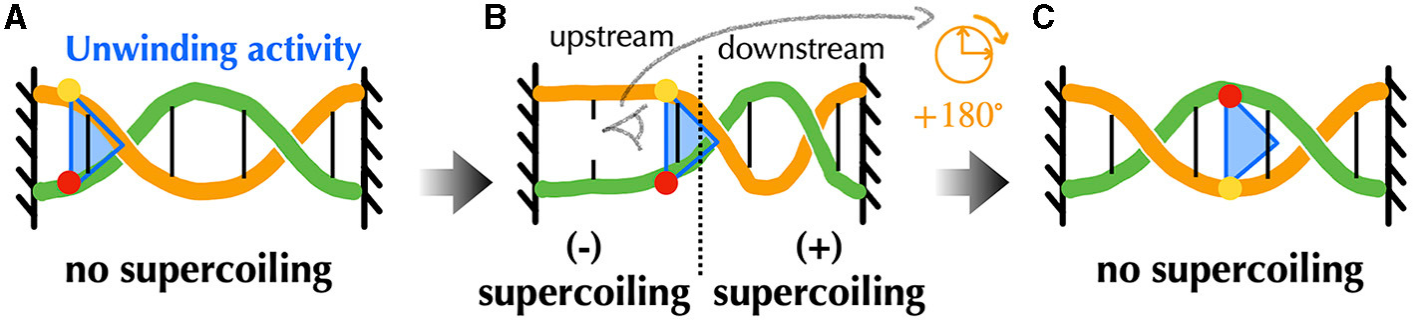
Schematic representation of torsional stresses generated during translocation of a DNA unwinding machine, starting from a situation with no supercoiling. Four base pairs (vertical lines) are indicated to facilitate reading. **(A)** The DNA ends are prevented from rotating, mimicking the effect of a topological barrier. Two extreme possibilities can then be considered. **(B)** If the unwinding machine does not rotate around the DNA, it behaves as a topological barrier and the double helix becomes increasingly overwound downstream and underwound upstream, respectively generating positive and negative supercoiling. The latter can lead to DNA denaturation, as indicated by the breaking of the base pair. **(C)** If the unwinding machine freely rotates around the DNA, the machine rotates clockwise while advancing along the undeformed right-handed DNA double helix.

Before providing details about topoisomerases, let us explicit the nature of the torsional stresses they relax during gene transcription and DNA replication. Namely, in both processes, associated macromolecular complexes including the RNA and DNA polymerases locally open bacterial DNA and proceed along it in a specific direction. Multiple protein complexes are bound to this DNA, and the expected situation *in vivo* is that of a chromosome organized into DNA domains whose ends are prevented from rotating by topological barriers (Liu and Wang, 1987) (see Section 5 for details). Consider, in this case, a piece of DNA such that the Watson and Crick strands of the double helix are held in a rotationally fixed position at the borders (Figure 2A). Just as in a circular molecule, these constraints impose the conservation of the linking number between the two strands. Consider, then, an idealized machine locally opening the DNA and advancing along it (Figures 2B, C). To the extent that the local opening is associated with a local unwinding of the strands (not represented in Figure 2 for clarity), the conservation of the linking number implies that the remaining double helical parts have to overwind in compensation. In this context, the torsional stresses induced by the progressing machine depend on whether it can freely rotate around the DNA (Figure 2C) or not (Figure 2B). In the former case, the machine rotates clockwise while advancing along the right-handed DNA double helix and no additional torsional stresses are exerted beyond those due to the initial opening. In the latter case, the double helix becomes increasingly overwound downstream and underwound upstream. This means that the number of base pairs per helix turn decreases or increases correspondingly. The progression thus induces respectively positive downstream and negative upstream *twin* DNA supercoiling (Liu and Wang, 1987), although *no net overall supercoiling* has been introduced.

**Figure 3:**
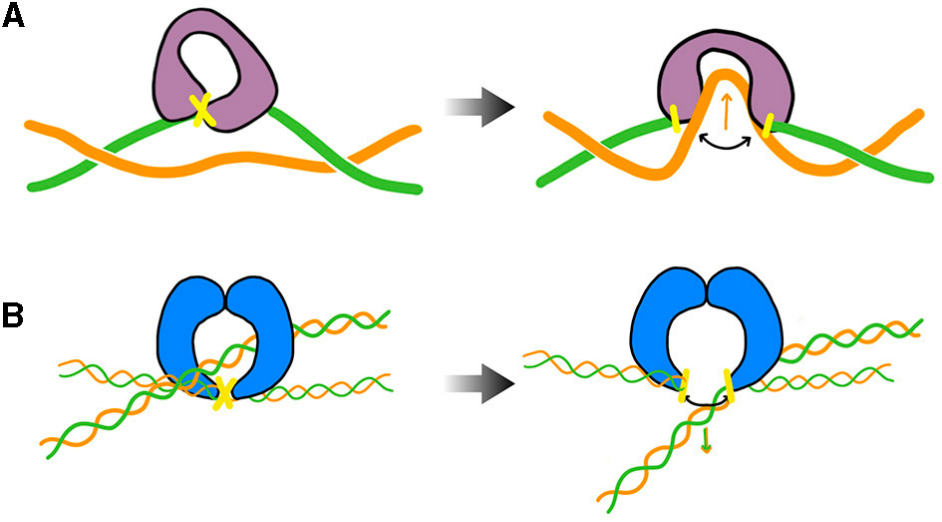
Cartoon of the main step responsible for the change of the DNA's linking number during the enzymatic cycle of prokaryotic Topo I (TopA, equivalently) and DNA gyrase— DNA cuts are indicated in yellow. **(A)** TopA scenario (adapted from Wang, 2002; McKie et al., 2021): the enzyme cuts a single strand (class I topoisomerase) of DNA and makes the other strand pass through the cut before DNA is re-ligated (type IA). Note that the prokaryotic Topo I makes the twist change by a single unit, whereas the eukaryotic Topo I makes the twist change by multiple units by allowing rotation of the uncut strand (type IB). **(B)** DNA gyrase scenario (inspired from Nöllmann et al., 2007): the enzyme cuts both strands of one DNA duplex (class II topoisomerase) and makes another duplex pass through the cut before DNA is re-ligated. The blue shapes indicates a dimer of GyrB, omitting specific structural details. The complete complex involves an additional dimer of GyrA. More detailed information on these structures and enzymatic cycles can be found in McKie et al. (2021).

During transcription elongation *in vivo*, although direct evidence is currently lacking, numerous experiments suggest that an RNA polymerase (RNAP) generally undergoes minimal rotation around DNA (see Section 5). Consequently, it generates both negative and positive supercoiling behind and ahead of it. This supercoiling implies a restoring torque from DNA acting on the RNAP. Without the release of this torque, the RNAP would eventually stall (Ma and Wang, 2014) and transcription might terminate. This issue is resolved by topoisomerases. Specifically, evidence in various mesophilic bacteria points to a major role of Topo I and DNA gyrase, which are able to respectively remove negative and positive supercoiling upstream and downstream the RNAP. The enzymatic reaction of the prokaryotic Topo I involves cutting one strand of the DNA duplex (class I topoisomerase) and making the other strand pass through the cut (Figure 3A). This process introduces positive (+1) *twist* to the DNA molecule, which relieves the torsional stress associated with negative supercoiling. DNA gyrase, on the other hand, can adopt multiple modes of action (Nöllmann et al., 2007). In all cases, its enzymatic reaction involves cutting both strands of the DNA duplex (class II topoisomerase) and making another duplex pass through the cut (Figure 3B). In ATP-consuming modes, via this process and an initial chiral wrapping of DNA (Basu et al., 2018), DNA gyrase introduces negative (–2) *writhe* to the DNA molecule, which can then be rapidly converted into negative twist to alleviate the torsional stress associated with positive supercoiling.

During DNA replication *in vivo*, numerous experiments also suggest that DNA polymerase rotates while advancing along the unreplicated DNA. This rotation nevertheless appears not be sufficiently rapid to prevent the accumulation of torsional stress in front of the replication machinery. Just as in transcription, the main topoisomerase that resolves this issue is DNA gyrase. Importantly, the rotation of DNA polymerase gives rise to an additional specific topological stress during the replication process, resulting in the newly synthesized DNA molecules twisting around each other and forming super-helices called precatenanes (Section 6). The resolution of these precatenanes is primarily carried out by a class II topoisomerase known as Topo IV, whose exact mechanism of action *in vivo* is still debated (Section 6.1).

Altogether, while additional topoisomerases exist (Wang, 2002; Forterre et al., 2007; McKie et al., 2021), DNA gyrase, Topo I, Topo IV but also Topo III [a class I topoisomerase involved in decatenation of replicated DNA (McKie et al., 2021)] are considered as the most important topoisomerases in mesophilic bacteria. Notably, the average linking number of DNA in these bacteria has been shown to predominantly reflect the relative activity of Topo I and DNA gyrase only (Drlica, 1992; Rovinskiy et al., 2012) as well as Topo IV (Zechiedrich et al., 2000).

### 2.3. Supercoiling density and its measurement

What is the level of DNA supercoiling in bacteria? This simple question actually carries various subtleties related to measurement, particularly *in vivo* measurement. To comprehend this issue, let us briefly revisit the classical methodology used for supercoiling measurements in cells. First, it is important to note that DNA supercoiling *is not measured on chromosomes, but on plasmids*. The latter are small circular DNA molecules of about 2–5 kilobase pairs (kb) that coexist with chromosomes and can be easily extracted from cells to quantify their linking number. This quantification relies on the measurement of plasmid migration properties on gels as these are sensitive to the compaction status of plasmids and, hence, to their level of super-structuring. The tacit assumption that plasmids are good topological proxies for chromosomes is then justified by the fact that topoisomerases are expected to behave similarly on both chromosomal and plasmid DNA.

Next, to compare DNA supercoiling levels between different bacteria, it is useful and customary to define the supercoiling density σ. This value is equal to the relative difference between the measured linking number and the linking number for the mechanically relaxed state: $\sigma =\frac{\text{Lk}-{\text{Lk}}_{0}}{{\text{Lk}}_{0}}$. The supercoiling density thus indicates the relative over- or under-winding of a DNA molecule with respect to the winding of a relaxed molecule (Figure 1). Namely, if Lk < Lk_0_, the supercoiling density σ is negative and the molecule has typically fewer helices than the corresponding relaxed B-DNA molecule, meaning that DNA is underwound with more base pairs per turn. Inversely, if Lk > Lk_0_, σ is positive and DNA is overwound, with less base pairs per turn. Let us recall, nevertheless, that part of the difference in local helicities between a supercoiled molecule and its relaxed counterpart takes the form of super-structuring (Figure 1)—see Section 4 for further details.

Finally, in addition to being an indirect estimate of chromosomal supercoiling, reported values of supercoiling densities usually correspond to quantities that are averaged over a cell population. Assuming a homogeneous population, this is equivalent to averaging over time. In this context, the measured supercoiling densities have been found to be negative for mesophilic bacteria, with mean values not exceeding −0.1 (Bliska and Cozzarelli, 1987). Note that it has been argued that this negative supercoiling is maintained by a proper balance of topoisomerase activity in the context of the regulation of gene expression (Menzel and Gellert, 1983), as negative values tend to favor transcription initiation (Section 5).

Altogether, these considerations mean that chromosomes of mesophilic bacteria are *predicted to be underwound on average, i.e., along the genome and over time*. More precisely, using the definition of σ, the number of base pairs per helix turn in the absence of writhe, denoted *n*_σ_, verifies $\sigma =\frac{1/n_{\sigma }-1/n_{0}}{1/n_{0}}$, where *n*_0_ ≃ 10.5 is the corresponding number for torsionally relaxed B-DNA. Therefore, *n*_σ_ ≃ 10.5/(1+σ) such that, for a typical measured value of σ = −0.05 (Bliska and Cozzarelli, 1987), *n*_σ_ ≃ 11.1 base pairs.

Let us nevertheless finish by noting that recent molecular techniques associated with DNA sequencing, such as Psora-seq (Visser et al., 2022) or GapR-seq (Guo et al., 2021), have paved the way for estimating supercoiling levels along chromosomes. Results show in particular that genomic distributions reflect transcriptional activities. Models aiming at predicting, or simply explaining these profiles, thus need to be developed in the context of transcription, in particular by including the specific action of topoisomerases (Section 5).

## 3. Physical modeling of supercoiled DNA: fundamentals

If bacterial genomes are relatively small compared to those of eukaryotes, chromosomes comprising several million base pairs are nevertheless gigantic macromolecules with contour lengths in the mm range, whose shapes undergo permanent changes due to thermal fluctuations and the action of the molecular machinery living organisms have evolved to structure, transcribe and replicate the genome. In this section, we introduce the notion of physical modeling and explain how such an approach applied at a resolution of the atoms comprising the DNA molecule, although feasible in principle, face unsurmountable difficulties on the possibility of brute-force modeling such gigantic macromolecules. We then explain how successive approximations, also known as coarse-grained descriptions, can be considered by dropping more and more details of the molecule. We introduce more particularly the rod-like chain model (Vologodskii et al., 1992; Bouchiat and Mézard, 2000), which is the simplest model for studying the folding properties of supercoiled DNA. For more details we refer the reader to the Appendix, where we provide a more exhaustive introduction into the subject of physical modeling.

### 3.1. Atomistic modeling

The prototypical example for physics based modeling is the work by Newton, who defined an equation of motion (the acceleration of a body is equal to the ratio of the force acting on it and its mass) and the “force field” describing the gravitational interaction between massive bodies like the sun, the earth, and the proverbial apple. By solving these equations, Newton was able to explain that Kepler's laws of planetary motion in the sense that they emerge from this more fundamental description, which also describes the ballistic trajectory of a cannon ball on earth (Weinberg, 2015).

Conceptually, Molecular Dynamics Simulations (Frenkel and Smit, 2001; Karplus and McCammon, 2002; Brooks et al., 2009; Case et al., 2021) proceed on an atomic level along the same lines. It is often used in the framework of Statistical Mechanics (Section A2 of the Appendix) to explain or predict emergent macroscopic properties from the behavior of microscopic (e.g., atomic) states. The underlying equations of motions for the atoms are those of Newton, which are nowadays solved numerically for force fields modeling the bonded and non-bonded interactions between the atoms. The emergent properties for, say, a model of water are now phase diagrams or material constants like the viscosity describing the liquid phase. In principle, such molecular dynamics simulations are ideal tools for studying the complexities of biomolecular systems. With steady advances in available computer power and the performance of employed codes (Shaw et al., 2014; Abraham et al., 2015; Eastman et al., 2017; Phillips et al., 2020; Thompson et al., 2022), they provide an ever more powerful “computational microscope” (Lee et al., 2009; Dror et al., 2012) into biomolecular structures and processes. Of particular interest for this review is their ability to help rationalize the structural properties of supercoiled DNA molecules (Mitchell et al., 2011), that is, the different ways of distributing the linking number of a molecule between the twist and the writhe (Section 2). In particular, both cryo-electron microscopy (Irobalieva et al., 2015) and atomic force microscopy (Pyne et al., 2021) have revealed a diversity of spatial conformations significantly larger than that initially thought, as well as a systematic presence of sharply bent DNA and kinks. Molecular dynamics simulations could confirm this diversity and further highlight the mechanisms associated with the local deformation of DNA (Irobalieva et al., 2015; Pyne et al., 2021), such as the tendency of nucleobases located at sharp bends to adopt splayed configurations.

Extending the domain of application of molecular dynamics simulations, which currently concern molecules of a few hundred base pairs, to the bacterial scale is however not feasible with current technology. Namely, using a single GPU for a system composed of 10^6^ atoms, one can currently simulate on the order of 10 nanoseconds per day. While this allows reaching the microsecond scale in 100 days, simulating an entire 5 Mb long bacterial genome, which comprises on the order of 10^9^ atoms, over biologically relevant time scales remains elusive. For instance, simulating a 100-minute-long cell cycle would require the time elapsed since the extinction of the dinosaurs. Coarse-grained models (Saunders and Voth, 2013; Dans et al., 2016; Jewett et al., 2021), which consist of dropping fine details below a given resolution to build simpler descriptions that capture properties above this resolution, are thus inevitably needed to rationalize and predict the structuring properties of DNA *in vivo*.

### 3.2. DNA fiber models

**Figure 4:**
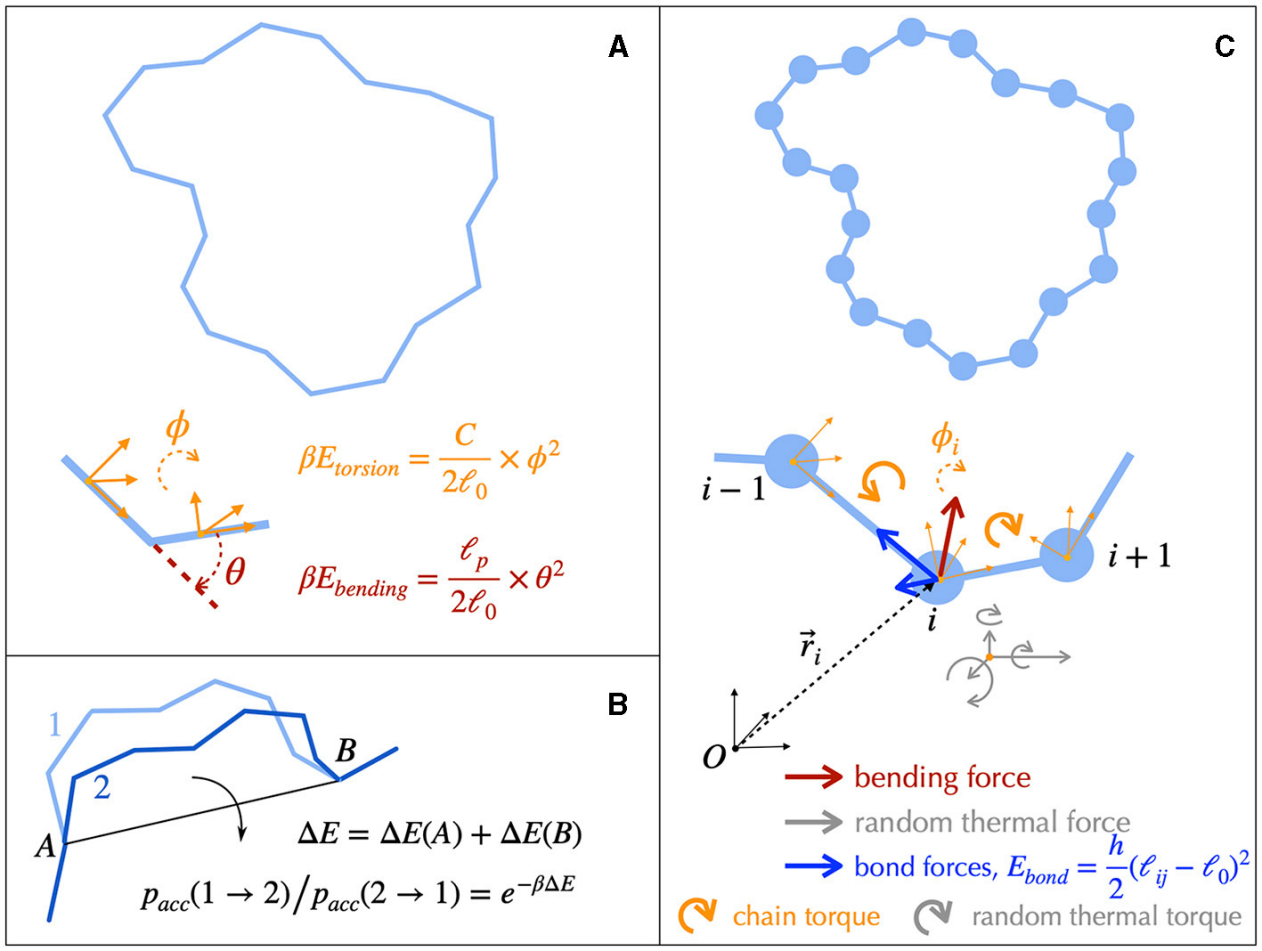
Fiber models of DNA and numerical simulations. **(A)** The simplest physical model of supercoiled DNA simplifies the DNA as a rod-like chain discretized into segments of a certain length ℓ_0_. The model involves only two independent parameters, the bending and the torsional moduli, which are respectively given by ℓ_*p*_/ℓ_0_ and *C*/ℓ_0_. ℓ_*p*_ is known as the bending persistence length and *C* as the torsional persistence length. The associated energies are harmonic potentials of the bending angle θ and of the torsion angle *ϕ*, respectively. The latter quantifies the rotational variation of the frames associated to each segment, a frame representing an orthonormal basis as indicated here by the orange vectors. $\beta^{-1}=k_{B}T$ defines the thermal energy. **(B)** A typical Monte Carlo simulation of the rod-like chain consists in iteratively rotating random groups of contiguous segments. The rotation of a specific group of segments is performed around the axes joining their flanking articulation points (*A* and *B*). It is accepted depending on the associated variation of energy Δ*E*, which involves only the articulation points, according to a probability rule (equation) that ensures reaching thermodynamic equilibrium at long time. **(C)** DNA dynamics can be simulated using Brownian Dynamics methods. To this end, DNA is modeled as beads on a string. At each time step, the motion of each bead is updated according to its equation of motion, which involves frictional forces and torques from the solvent (not shown), forces and torques coming from the neighboring connected beads, and random forces and torques (thermal noise from the solvent).

Structural details of DNA can be coarse-grained to build single-nucleotide resolution polymer models (Harris, 2006; Ouldridge et al., 2011; Manghi and Destainville, 2016). Simulating such models is nevertheless still limited to less than 1 kb long molecules, calling for less resolved models to investigate structuring properties above the gene scale. In this regard, rigid base (Lavery et al., 2009; Gonzalez et al., 2013) or base-pair (Olson et al., 1998; Becker et al., 2006; Becker and Everaers, 2009; Lavery et al., 2009) models of B-DNA allow to preserve the sequence-dependent structure and elasticity of the canonical double-helix. Further coarse-graining (Becker and Everaers, 2007; Petkeviciūtė et al., 2014; Gutiérrez Fosado et al., 2023) leads to tens-of-base-pairs resolution fiber models of DNA (Figure 4), which still preserve the microscopic mechanical properties of DNA. These fiber models have been used to address the properties of DNA molecules up to several tens kb (Section 4.2). The rod-like chain model (Vologodskii et al., 1992; Bouchiat and Mézard, 2000) is a prototypical example in which DNA is modeled as a series of articulated rigid segments (Figure 4A). The relative orientation of consecutive segments is constrained by two parameters, the bending and torsional moduli, which quantify the resistance of DNA to bending and torsion, respectively. Importantly, fiber models neglect the specific structure of the double helix itself. As a consequence, they necessitate the inclusion of an effective treatment for conserving the linking number (Section A2 of the Appendix).

### 3.3. Numerical simulations

**Figure 5:**
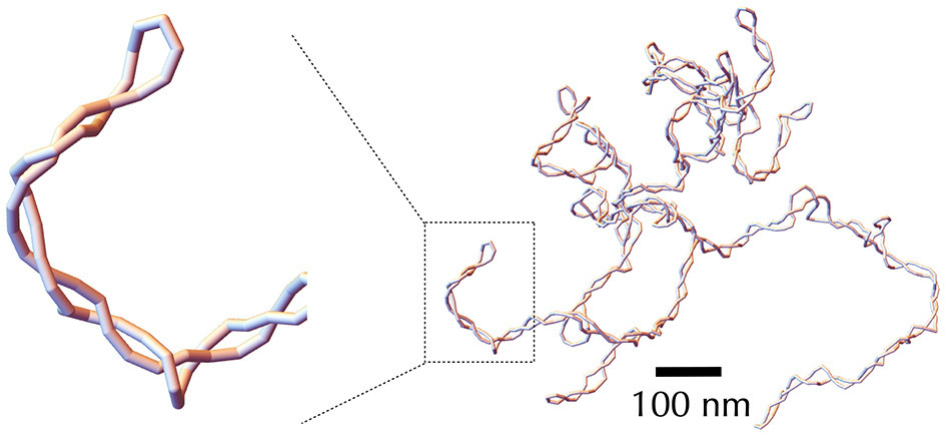
Snapshot of a typical DNA conformation obtained through Monte Carlo simulation using the self-avoiding rod-like chain model, with a negative supercoiling density *σ* = −0.06. The DNA molecule considered here is 30 kb long, with each segment containing 30 bp. The leftmost panel provides a zoomed-in view of a plectonemic super-structure, emphasizing the discrete nature of the segments.

Solving analytically the simplest model as the rod-like chain model by predicting for instance the spatial extension of the molecule leads to unsurmountable difficulties. Anticipating phenomena where volume exclusion plays an important role such as in the presence of plectonemic DNA is also known to be a difficult task, although phenomenological approaches based on thermodynamic arguments have been proved to be particularly insightful (Section A3 of the Appendix). Numerical simulations of polymer chains are thus often necessary to investigate the folding properties of DNA fiber models. In this regard, equilibrium properties can be studied with the help of Monte Carlo simulations (Frenkel and Smit, 2001), which allow to explore the space of possible DNA conformations often in an efficient way, with the help of a non-physical random dynamics (Figure 4B). Dynamical properties can be studied using Brownian Dynamics simulations (Allison and McCammon, 1984; Chirico and Langowski, 1992). To this end, the DNA chain is described in terms of beads (Chirico and Langowski, 1994; Jian et al., 1997) (Figure 4C) and its motion is simulated by considering the equations of movement for the beads. Namely, Brownian dynamics simulations assume that each DNA bead experiences a combination of friction and (correlated) random forces and torques coming from the solvent (cytoplasm) plus a combination of forces and torques coming from the translational and rotational motions of the connected neighbor beads along the chain (Figure 4C). Applied to the rod-like chain model where the self-avoidance properties of the DNA chain is also considered, these simulations show that the three-dimensional folding induced by DNA supercoiling is indeed of a plectonemic type (Vologodskii and Cozzarelli, 1994) (Figure 5).

## 4. Structural predictions from equilibrium fiber-like models

Molecular tools have been developed to probe the topology of DNA *in vivo* at multiple scales (Lagomarsino et al., 2015), with the recent possibility of obtaining information on the distribution of torsional stress along the genome (Guo et al., 2021; Visser et al., 2022). Yet, the *in vivo* occurrence and permanence of associated structural phenomena remain poorly quantified. Difficulties lie both in the difficulty of measuring supercoiling densities (Section 2.3) and in the small size of the structures involved (of the order of the nm). Many modeling questions have thus revolved around predicting the relative proportion of local deformation of the double helices and of super-structuring. Equilibrium Statistical Mechanics (Section A3 of the Appendix) has played a major role in this matter. In the following, we discuss in more details both outcomes of these approaches and their relevance for *in vivo* situations, which is a consequence of the often “near-equilibrium” nature of phenomena. To this end, we first present one-dimensional models aiming at specifically capturing the local deformations of the DNA duplex. We next present three-dimensional models aiming, *in fine*, at capturing both the local deformations of the DNA helix together with the overall folding of the molecule. Finally, we discuss how these models have recently been used to provide novel physical insights into the question of the nature of the topological barriers that have been detected *in vivo*.

### 4.1. One-dimensional models: predicting denaturation bubbles and other non-B DNA motifs

The intensity of supercoiling-induced mechanical stress depends on the local DNA sequence. As a consequence, various phenomena can take place at specific locations along the genome. These include DNA denaturation as shown by the pioneering work of Vinograd and his collaborators in the 1960s (Vinograd et al., 1968), generation of DNA forms alternative to B-DNA (Mirkin, 2008) and generation of alternative secondary DNA structures such as cruciforms (Mizuuchi et al., 1982). Importantly, some of these structural motifs have a functional role, making the *physical prediction* of their occurrence and distribution along the genome an important *biological problem* (Du et al., 2013).

**Figure 6:**
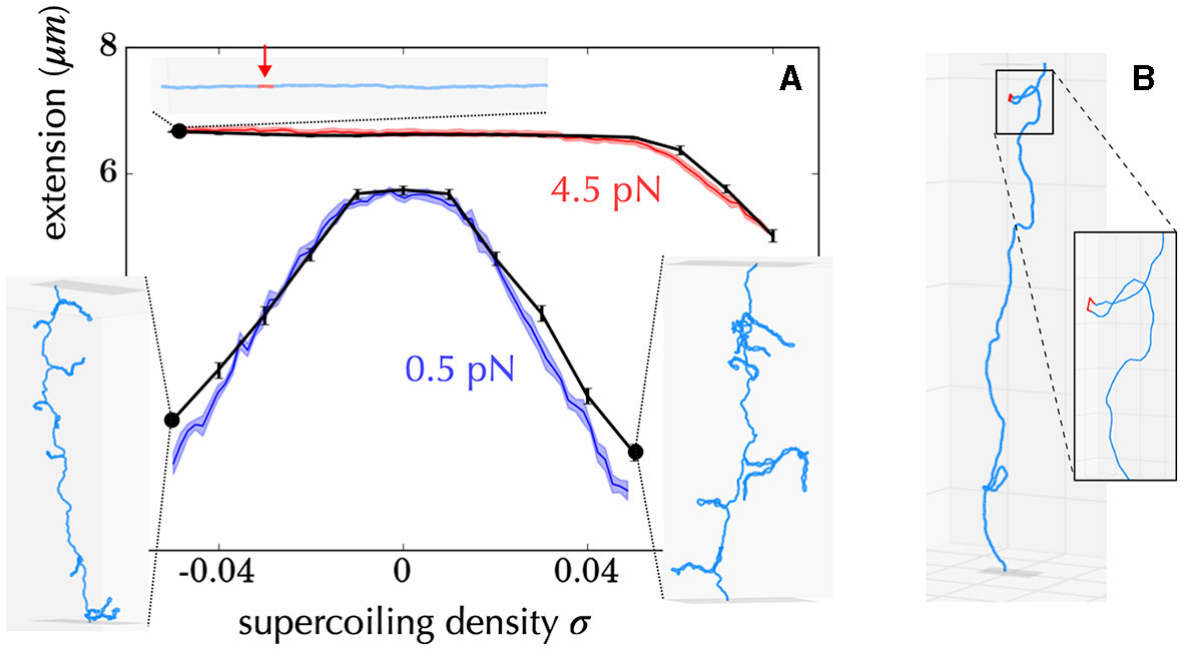
Example of diversity of structures obtained using a coarse-grained model of DNA at a resolution of 10 bp, including the possibility of forming alternative DNA structures such as denaturation bubbles (Lepage and Junier, 2019). **(A)** Comparison between experiments (Vlijm et al., 2015) (colored curves) and simulations (black curves) for a 21 kb long molecule manipulated by a magnetic tweezer. The *x*-axis indicates the imposed supercoiling density on the molecule, and the *y*-axis shows the measured extension of the molecule. The experiments were conducted at two forces (0.5 pN in blue and 4.5 pN in red). The inner panels show the typical conformations of the molecule obtained in the simulations for different experimental parameters (black dots). For example, the top panel indicates that when the molecule is stretched at 4.5 pN and undergoes a negative supercoiling of ~ −0.04, a denaturation bubble forms (indicated in red). The other conformations indicate the presence of plectonemes. **(B)** For some force and supercoiling density, conformations can display denaturation bubbles (in red) located at the apex of plectonemes. These were initially predicted to occur using a coarse-grained polymer model of DNA at the nucleotide level (Matek et al., 2015).

How are Statistical Mechanics models built to address the problem of the tendency of a given subsequence of DNA to denature or form alternative forms? First, they most often neglect the effects of writhe, which is similar to assume a stretching force of a few pN (Figure 6), so that the problem becomes one-dimensional (Fye and Benham, 1999). In doing so, analytical calculations are possible, making it possible to establish mathematical relationships between observables (i.e., measurements performed on the system) and system parameters (e.g., supercoiling level). It is then possible to predict behaviors without resorting to simulations which are often time-consuming and limited from the viewpoint of exhaustivity. Second, most approaches assume that supercoiling constraints are relaxed much faster than they are produced (*near-equilibrium condition*). This hypothesis is justified, for example, in the case of transcription, whose initiation step requires the formation of a DNA denaturation bubble (Murakami and Darst, 2003; Mejía-Almonte et al., 2020). Namely, the twist and writhe relaxation times (below 1 ms) for a 10 kb long molecule are typically four orders of magnitude smaller than the time for synthesizing a 1 kb long messenger RNA (≥10 s) (Joyeux and Junier, 2020; Fosado et al., 2021; Wan and Yu, 2022) and one order of magnitude with respect to the time for synthesizing a single base pair. Thus, questions concerning the energy required to denature DNA have been systematically addressed in the context of the equilibrium statistical mechanics of one-dimensional systems (Benham, 1979; Fye and Benham, 1999; Jost et al., 2011). In particular, efficient semi-analytical approaches allow to predict the most probable sites of denaturation at the scale of a genome (Jost et al., 2011; Jost, 2013).

Despite simple assumptions with respect to the complexity of *in vivo* phenomena, including the neglect of super-structuring, these equilibrium one-dimensional approaches have been shown to be sufficiently predictive to be used, for example, in the analysis of the sensitivity to supercoiling of transcription initiation (Forquet et al., 2021), in accord with the necessity of DNA to denature at the promoter (see Section 5 for insights). This suggests that strong deformations of the double helix is often dominant *in vivo* and, hence, that forces on the pN range are expected to act on bacterial DNA (Strick et al., 2003). Along the same line, these approaches have been used to predict the appearance and location of non-B DNA motifs (Wang et al., 2004; Du et al., 2013), which appears to reflect the role of supercoiling in the regulation of transcription (Du et al., 2013).

### 4.2. Three-dimensional structuring: from the first observations to the first polymer models

In addition to demonstrate that supercoiled DNA denatures, Vinograd and his collaborators used electron microscopy experiments to reveal, for the first time, the capacity of (viral) circular DNA molecules to form super-structures (Vinograd et al., 1965; Vinograd and Lebowitz', 1966). Notably, they observed “plectonemes,” while the term would be only coined in the late 1980s (Vologodskii et al., 1992). Remarkably, they attributed these super-structuring properties to invariant topological properties of circular molecules: “In closed double circular DNA, the number of degrees of angular rotation of one strand around the other is invariant” (Vinograd and Lebowitz', 1966). Several years later, in the 1970s, pioneering electron microscopy experiments revealed that the bacterial chromosome extracted from *Escherichia coli* cells was also made of plectonemes (Delius and Worcel, 1974; Kavenoff and Bowen, 1976) but also of numerous loops (Delius and Worcel, 1974). In 1990, *in vitro* experiments, still visualized by electron microscopy, showed for *in vivo* relevant values of supercoiling density a systematic tendency of bacterial DNA to form plectonemes at small scales and trees at large scales (Boles et al., 1990).

These results raised fundamental questions, starting with the physical mechanisms behind the formation of plectonemic structures. In particular, since an excess of writhe could manifest itself in the form of solenoids, how to explain the prevalence of plectonemes? This question remained unanswered for many years before being partially solved in the early 1990s with the help of the first polymer models of supercoiled DNA at a resolution of a few tens base pairs (Klenin et al., 1991; Vologodskii et al., 1992). These models, which are still at the basis of current works, account for the electrostatic repulsion of DNA (self-avoidance), the energies of DNA bending and torsion, which result from a coarse-grained description of DNA that neglects fine atomic details, as well as the global constraint of the conservation of the linking number (Section 3). In 1994, the question was definitively resolved by Marko and Siggia on the basis of a quasi-analytical solution of a phenomenological equilibrium thermodynamics description of these microscopic models (Marko and Siggia, 1994), showing that under physiological conditions of temperature, salt and supercoiling density, plectonemes are thermodynamically favored compared to solenoids. The reason lies in the “large” energy of bending of solenoids, which can be reduced drastically in plectonemes while keeping similar torsional stresses (Marko and Siggia, 1994). Single-molecule magnetic tweezers experiments combined with fluorescent labeling of DNA (van Loenhout et al., 2012) and polymer simulations (Lepage et al., 2015) have then shown that the length of plectonemes *in vitro* are on the order of 1 kb. Electron microscopy (Boles et al., 1990) and statistical mechanics of plectonemes (Marko and Siggia, 1995; Barde et al., 2018) also revealed a diameter of the plectoneme varying between ≃30 nm at σ≃−0.025 and ≃5 nm at σ≃−0.1. Finally, due to the entropic contribution of branches, Marko and Siggia further showed that plectonemic structures become branched and form trees at large scales (Marko and Siggia, 1995), rationalizing both experiments (Boles et al., 1990) and numerical simulations (Vologodskii et al., 1992).

### 4.3. A simulation toolbox to anticipate structuring properties of bacterial DNA

The pioneering work of Vologodskii and his collaborators in the early 1990s, which focused on the development of Monte Carlo simulations (Section A5) for topologically constrained polymer chains (Vologodskii and Cozzarelli, 1994), sparked intense and ongoing research on the thermodynamic properties of supercoiled DNA at the scale of a few kb, typically up to a few tens kb (see Figure 6 for an example). The self-avoiding rod-like chain model (Vologodskii et al., 1992) (Figure 4), also known as the twistable worm-like chain model (Nomidis et al., 2019), is typical of this approach and has been instrumental in analyzing the equilibrium folding properties of both positively and negatively supercoiled DNA molecules without strong deformation of the B-DNA double helix. These properties include molecular extensions (Vologodskii and Marko, 1997), torques (Schöpflin et al., 2012; Lepage et al., 2015), and conformation details of super-structures (Vologodskii et al., 1992; Bednar et al., 1994; Klenin et al., 1995). The models can be extended to include DNA denaturation and the formation of alternative forms that occur at high negative supercoiling levels (Lepage and Junier, 2019). Brownian dynamic simulations of supercoiled DNA (Figure 4, Section A5) were also developed in the early 1990s by Langowski and his collaborators, enabling the study of the dynamical properties of DNA loci (Chirico and Langowski, 1994).

This toolbox of polymer simulations has been used for more than 30 years not only to rationalize experimental but also to anticipate possible non-trivial properties of supercoiled DNA. An illustrative example comes from an early study by Langowski and collaborators. Namely, their Brownian dynamics simulations predicted in the late 1990s that plectonemes should move along the DNA not only through (slow) diffusion but also by disappearing at one location to reappear at a distant location along the DNA (Chirico and Langowski, 1996). This “hopping” type of motion was observed years later in fluorescent-labeling single-molecule experiments for supercoiled DNA stretched by pN range forces (van Loenhout et al., 2012) and, hence, is expected to occur as well *in vivo* – plectoneme hopping in van Loenhout et al. (2012) could be distinguished by the concomitant disappearance of a fluorescence spot (associated with the high density of a plectoneme) and appearance of another spot along the molecule. Brownian dynamics simulations further revealed, for molecules of a few kb, that loci tend to make contacts through intra-plectoneme slithering (secondary type of contacts) rather than through inter-plectoneme random collisions (tertiary type of contacts) (Huang et al., 2001)—tendency that may be reinforced by the hopping motion of plectonemes. The genomic range for which secondary contacts are expected to be more frequent than tertiary contacts *in vivo* nevertheless remains open. From a modeling viewpoint, this would require in particular to properly investigate finite size effects knowing that the size of molecules in simulations are at most on the order of a few tens kb, i.e., two orders of magnitude smaller than e.g., the chromosome of *E. coli*.

### 4.4. Topological barriers: insights from Physics

Contrary to a naive vision of a topological constraint (the linking number) acting on the chromosome as a whole, molecular genetics experiments, genetic recombination assays and electron microscopy of isolated chromosomes have revealed that the genomes of *E. coli* and *Salmonella* are actually organized into topologically independent domains whose size is on the order of 10 kb (Postow et al., 2004; Deng et al., 2005). Comparative genomics further predicts this organization to be ubiquitous in bacteria and to be associated with the basal coordination of transcription (Junier and Rivoire, 2016; Junier et al., 2018). Yet, the nature of the topological barriers associated with this partitioning have remained highly debated. In this regard, polymer simulations have provided insights into the possible implication of several factors.

**Figure 7:**
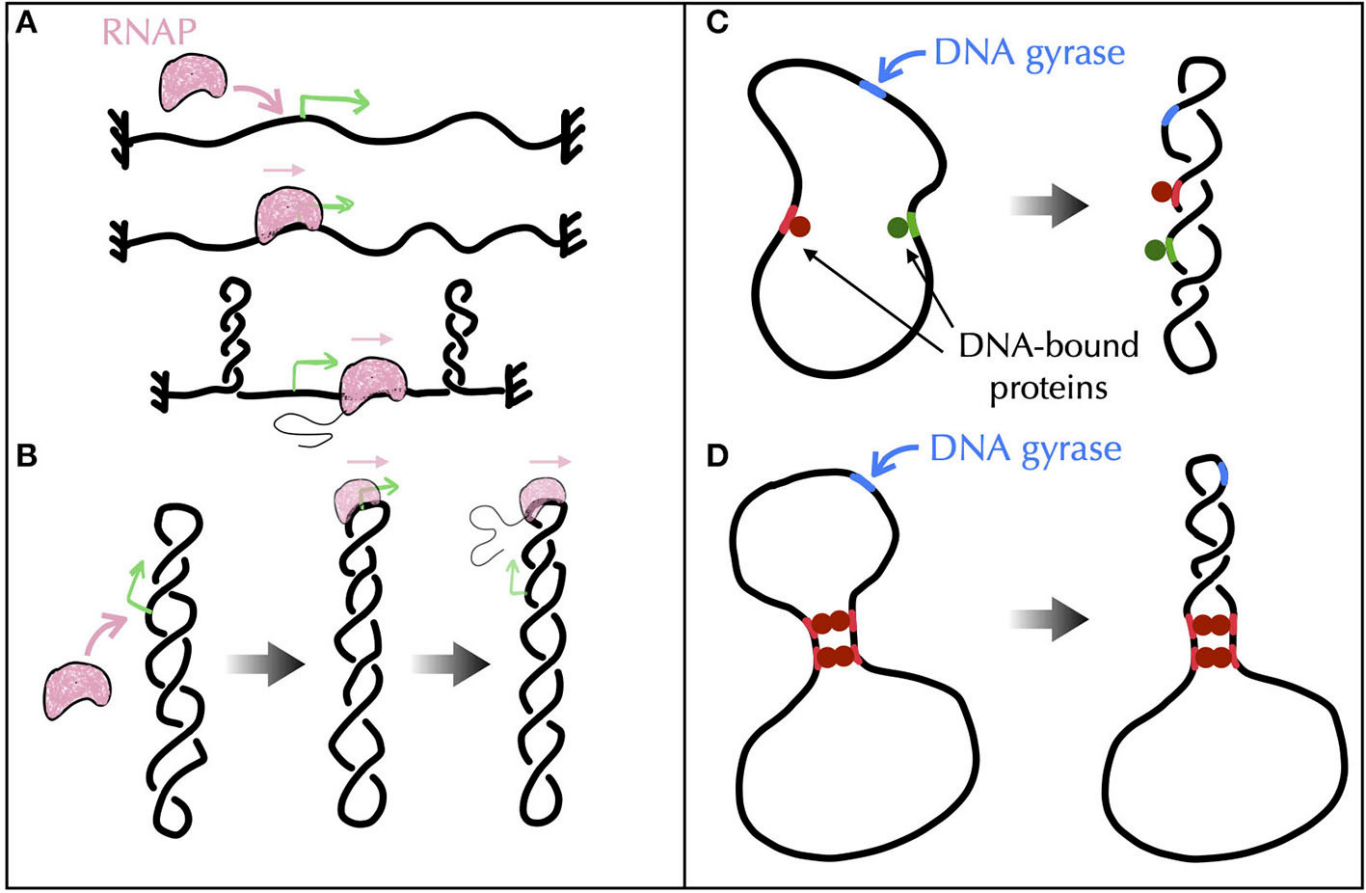
Models of topological barriers as suggested by polymer simulations of supercoiled DNA in the presence of various proteins and enzymes. **(A)** Simulations of an active process where an RNAP generates supercoils in a topologically constrained domain (Fosado et al., 2021) suggest that the RNAP itself can function as a topological barrier. In (Fosado et al., 2021), the generated supercoils would indeed relax under the form of plectonemes that occur “far” from the RNAP. The RNAP thus prevents the mixing of the topological properties of the upstream and downstream DNA regions. **(B)** Other studies (Racko et al., 2018; Joyeux, 2022) have reported a tendency for a translocating RNAP to localize at the apex of plectonemes. In this case, the RNAP does not act as a topological barrier since the upstream and downstream DNA segments are intermingled. **(C, D)** To evaluate the potential of DNA-bound proteins (green and red disks) to act as topological barriers, a possible experimental setup consists in considering a plasmid with a strong gyrase binding site (in blue) and in checking whether gyrase activity at this site causes the entire plasmid, or only the region flanked by the binding sites of the proteins, to adopt a plectonemic super-structure (Leng et al., 2011). **(C)** Numerical simulations show that proteins that do not bridge DNA, even if they impede twist diffusion, are incapable of acting as topological barriers (Joyeux and Junier, 2020). **(D)** Proteins that block the diffusion of both twist and writhe, as in the presence of multiple successive bridges (Leng et al., 2011), effectively operate as topological barriers (Joyeux and Junier, 2020).

First, experimental results obtained *in vivo* from genetic recombination assays sensitive to the formation of plectonemes (Higgins et al., 1996) strongly suggest that transcribing RNAPs behave as such topological barriers (Deng et al., 2004; Higgins, 2014). A possible rationale could come from active processes, i.e., from situations far from equilibrium (Section A4 of the Appendix). Namely, recent Brownian dynamics simulations have shown that the DNA supercoiling introduced by a transcribing RNAP might relax under the form of plectonemes that form far from the RNAP (Fosado et al., 2021) (Figure 7A). The absence of any plectoneme embedding the RNAP is indeed in accord with the capacity of this RNAP to block the diffusion of writhe. Yet, other modeling works have reported the tendency for a transcribing RNAP to locate at the apex of plectonemes (Racko et al., 2018; Joyeux, 2022) (Figure 7B), which is in opposition with its functioning as a topological barrier. The fundamental reason for the difference between the outcomes of these far from equilibrium models remain to be elucidated. Nevertheless, it is worth noting that the apical localization of plectonemes is consistent with previous *in vitro* experimental results (ten Heggeler-Bordier et al., 1992). Moreover, experimental studies indicate that a transcribing RNAP enhances the flexibility of DNA (see references in ten Heggeler-Bordier et al., 1992), while modeling studies have shown that the most flexible part of DNA tends to preferentially localize at the apex of plectonemes (Matek et al., 2015; Lepage and Junier, 2019) (Figure 6B).

Second, *in vitro* experiments combined with genomic analyses of protein binding sites suggest the participation of certain nucleoid associated proteins such as H-NS (Hardy and Cozzarelli, 2005; Noom et al., 2007). H-NS is indeed able to bridge DNA to form loops (Dillon and Dorman, 2010) and, as demonstrated in the case of LacI, GalR or λ O (Leng et al., 2011), these loops may define topological domains. The problem, then, is to identify the conditions DNA-bridging proteins must follow to be able to topologically insulate a genomic domain from its neighbor. In this regard, recent Brownian dynamics simulations have shown that not only bridges must block the diffusion of twist but they must also prevent DNA segments to rotate with respect to each other, i.e., they must block the diffusion of writhe, too (Joyeux and Junier, 2020) (Figures 7C, D).

How, then, to systematically test the ability of DNA-bridging proteins to create topological barriers? Experimental insights for the transcription factors LacI, GalR, or λ O have already been provided. The method consisted in combining biochemical techniques and atomic force microscopy to study folding properties of plasmids both in the presence of multiple binding sites of such proteins and under the action of DNA-nicking and gyrase activities (Leng et al., 2011). An interesting alternative approach could consist in exploiting fluctuation properties of supercoiled molecules. Specifically, the variance of the extension of a molecule, as a function of both its supercoiling density and the intensity of a stretching force acting on it, can be accurately predicted using a phenomenological approach (Skoruppa and Carlon, 2022). Next, while the average extension can be shown to be insensitive to the presence of a bridge within the plectonemes, the variance depends on the location of the bridge (Vanderlinden et al., 2022). In a proof of concept study, this property has been utilized to experimentally identify the position of topological barriers created by two-site-specific DNA restriction enzymes (whose cleavage was impeded). This was achieved by combining single-molecule experiments, Monte Carlo polymer simulations of supercoiled DNA, and analytical approaches (Vanderlinden et al., 2022).

### 4.5. The need for further coarse-graining the DNA fiber models

At a larger scale, can fiber models of DNA be used to simulate the folding of an entire bacterial chromosome? Supposing that thermodynamic equilibrium is relevant for the large scale organization of chromosomes, which should be the case for sufficiently slow cell growth, the question at hand is how long a simulation must run to reach thermodynamic equilibrium. To that end, we can consider the most effective Monte Carlo methods for forming and equilibrating supercoiled DNA structures, which involve chain deformations that are particularly well-suited to relaxing plectonemic structures (Liu and Chan, 2008). Simulations suggest that the characteristic number of iterations required to reach equilibrium in this context is of the order of the chain length (*L*) (Liu and Chan, 2008). Suppose, then, that the topological constraint of the conservation of the linking number is implemented locally (Carrivain et al., 2014; Lepage and Junier, 2017), simulations show that *K* elementary Monte Carlo moves (whose subchain sizes range from 1 to *L*) take a CPU time that scales as *K* × *L*^1.2^ (Lepage et al., 2015). Assuming that this time can be reduced to *K* × *L* (the exponent 1.2 reflects the management of the self-avoiding constraint), since *L* moves are necessary to reach equilibrium, the characteristic simulation time for the most efficient simulations should scale as *L*^2^—note that these simulations are challenging to parallelize due to non-trivial self-avoidance constraints (Krajina and Spakowitz, 2016).

Knowing that it takes about 5 hours on a 3.5 Ghz processor to reach equilibrium for a chain of 20 kb when the supercoiling density is not too intense (e.g., for σ = −0.03) (Lepage et al., 2015), the time to reach equilibrium for a ~500 kb long genome, such as the JCVI-syn3A synthetic minimal genome for which Hi-C data is available (Gilbert et al., 2021), is of the order of 5 × (500/20)^2^≃3000 hours, or approximately 130 days. For *E. coli*, the time is approximately 35 years. To scale up to chromosomes, particularly those with a length of a few Mb as that of *E. coli*, coarser-graining methods that neglect the details of plectonemes are thus necessary. In Section 8, we discuss two main types of models resulting from these procedures: trees and bottle brushes.

## 5. Supercoiling constraints and transcription

**Figure 8:**
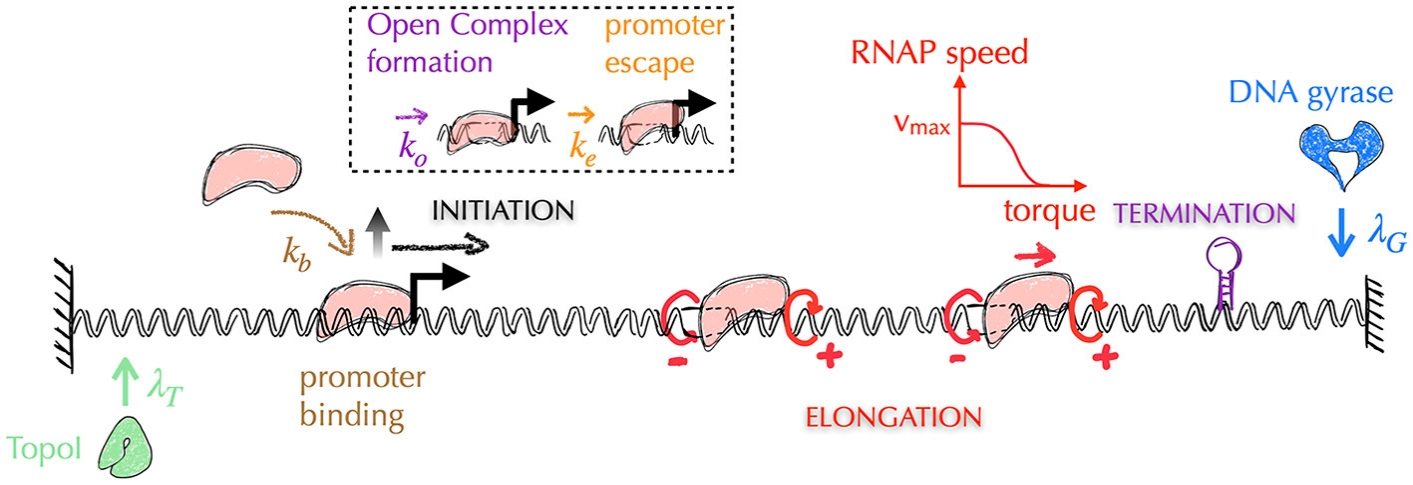
Typical architecture of physical models of gene transcription—for simplicity, we omit the representation of mRNAs. The most recent models of gene transcription are based on the biological principles of the twin transcriptional loop (TTL) scenario. In this scenario, a transcribing RNAP (in pink) does not rotate, generating on each side torsional stresses in the form of torques (circular red arrows, with the sign of the supercoils indicated by ±)—note that the supercoils generated between two successive RNAPs tend to cancel each other. Transcription is then usually divided into three sub-processes: initiation, elongation and termination. Elongating RNAPs are considered to act as topological barriers and the generated torques are estimated from the corresponding supercoiling values (Marko, 2007). In all models, the speed of RNAPs is highest when the torques are zero and decreases as the torque values increase. Depending on the model precision, the removal of supercoils by topoisomerases is considered either globally or locally. In the latter case, as shown in the figure, the models incorporate the distinct activities of Topo I and DNA gyrase, which act preferentially upstream and downstream of the gene, respectively (see main text). The initiation process can be further divided into multiple stages, including the promoter binding step and the subsequent steps that lead the DNA-bound RNAP into the elongation stage. These steps encompass the formation of the open complex and the promoter escape. Elongation is typically modeled as a deterministic process, where the speed of RNAP is a function of the torque acting on it (red curve). The other stages are modeled as stochastic processes, where the corresponding rates (λ_*T*_, λ_*G*_, *k*_*b*_, *k*_*o*_, *k*_*e*_) are often unknown and are therefore subjects of investigation (see e.g., Boulas et al., 2023 for rates associated with topoisomerases activity). Finally, it should be noted that the question of the three-dimensional folding of DNA and its impact on the different stages of transcription is currently not considered in these models.

Awareness of the central role of DNA supercoiling in transcription dates from the 1970s (Wang, 1974), with the seminal work of James C. Wang, who discovered the first topoisomerase (Wang, 1971) known today as Topo I—DNA gyrase was discovered 5 years later (Gellert et al., 1976). In particular, Liu and Wang (1987) hypothesized that the most frequent situation in bacteria for a transcribing RNAP is to generate supercoiling stresses on each side of it because of the impossibility of the RNAP to rotate around DNA. More precisely, because the transcribing RNAP and its associated mRNA interact with other macromolecules (ribosomes, regulatory factors, other RNAPs or DNA itself through e.g., the formation of R-loops Thomas et al., 1976), the resulting macro-complex experiences torsional friction. This hinders the rotation of the RNAP around DNA. In addition, DNA itself interacts with various macromolecules (e.g., membrane Lynch and Wang, 1993, clusters of RNAPs Stracy et al., 2015), which is expected to hinder its global rotation, too. As a consequence of the difficulty of both RNAPs and DNA molecules to rotate and according to the topological considerations of Figure 2, Liu and Wang (1987) surmised that the transcription of genes most often generates negative and positive DNA supercoiling upstream and, respectively, downstream the transcribing RNAPs, which they demonstrated for a particular case on a plasmid (Wu et al., 1988). The corresponding *biological* model is known as the twin transcriptional-loop (TTL) model (Liu and Wang, 1987). It is nowadays at the foundation of all *physical* models of the interplay between transcription and DNA supercoiling (Figure 8). In the following, we thus explain the ingredients and outcomes of these models, and discuss the open problems to be solved.

### 5.1. On including topoisomerase activity in physical models: some numbers

First, can a model based on the interplay between only DNA and RNAPs capture quantitatively gene transcription? To answer this question, let us recall a few numbers associated with gene transcription in the most-studied bacterium. In *E. coli*, RNAPs transcribe at a rate between 25 and 100 bp.s^−1^, depending on the growth rate of the bacterium (Bremer and Dennis, 2008). In the extreme case of an absence of rotation of the RNAP, this means that the DNA unwinding associated with transcription generates between ~2 to ~10 positive (negative) supercoils per second upstream (downstream) the RNAP— considering one supercoil per transcription of ~10 base pairs or one turn of the DNA double helix. Considering the presence of topological barriers located at a distance of ~10 kb (~1000 supercoils) that prevent the dissipation of these supercoils (Postow et al., 2004), according to the TTL model, transcription activity is expected to make DNA supercoiling density σ vary by an amount of at least 0.01 every second on each side of the transcription complex. With respect to DNA, the effects of supercoiling become significant for |σ| = 0.01 and highly disruptive for |σ| = 0.1 (Strick et al., 2003). With respect to RNAPs, single-molecule studies have suggested that they stall *in vivo* for torques (Γ) on the order of 18 pN (Ma et al., 2019) or equivalently, |σ|≃0.06—using σ = Γ/*A* where *A* = 300 pN is an average of the values estimated from single-molecule experiments for the regime where plectonemes are present (200 pN) and for the regime where super-structuring is absent (400 pN) (Marko, 2007). RNAP translocation along the DNA can then resume only if the associated torques are released, which can occur *in vivo* through two mechanisms: (i) another RNAP compensates the supercoiling, which however does not solve the problem upstream and downstream the train of RNAPs; (ii) topoisomerases relax supercoiling. In other frequent situations such as those involving divergent genes, supercoiling densities may actually vary even more abruptly. Namely, for two divergent promoters separated by a distance of ≃ 200 bp, the transcription of the upstream gene would create a transitory barrier and the total variations of supercoiling would be on the order of 0.1 every second.

Altogether, these numbers show that topoisomerase activity is required for transcription to properly proceed as soon as the elongating complex slowly rotates around DNA. Actually, an often overlooked ingredient of the TTL model is the inclusion of topoisomerases. Liu, Wang and collaborators indeed demonstrated that Topo I and DNA gyrase were responsible for relaxing the upstream negative supercoils and the downstream positive supercoils, respectively (Wu et al., 1988). They then anticipated that for gene expression to be properly predicted, one would need to include the activity of these topoisomerases (Liu and Wang, 1987; Wu et al., 1988). 30 years later, not only experiments have convincingly demonstrated that gene context plays a role in gene expression as important as transcription factors (Yeung et al., 2017; Nagy-Staron et al., 2021; Scholz et al., 2022), but they have also corroborated the relevance of the TTL model and the *necessity* to consider Topo I and DNA gyrase to quantitatively apprehend transcription (Chong et al., 2014; Ahmed et al., 2017; Yeung et al., 2017; Kim et al., 2019; Rani and Nagaraja, 2019; Ferrándiz et al., 2021; Sutormin et al., 2022; Boulas et al., 2023).

### 5.2. Physical implementation of the twin transcriptional loop (biological) model

The most recent *physical* models of the TTL thus include the interplay between DNA, RNAPs and topoisomerases. In a nutshell, they consist in including altogether both stochastic and deterministic parts of the transcription process. That is, they include with different levels of precision a stochastic description of transcription initiation, a deterministic description of RNAP elongation (with the speed being a function of the torque acting on the RNAP), a deterministic description of termination and a stochastic description of the action of topoisomerases (Figure 8). They then make the assumption that any elongating RNAP behaves as a topological barrier, or that it can absorb part of the supercoiling by rotating. The motion of RNAPs is then described at a spatial resolution of typically less than a few tens base pairs. Associated torques can indeed vary dramatically as soon as the RNAP transcribes a few base pairs: for two consecutive RNAPs separated by e.g., 100 (500) base pairs, it only requires the transcription of one (five) base pair(s) to make the supercoiling density vary by an amount of ~0.01. None of the models yet include the explicit structure of DNA (see Section A5 of the Appendix for an explanation). They nevertheless display a rich phenomenology that still needs to be fully understood.

More precisely, using these models, research groups have endeavored to quantify the downstream accumulation of positive supercoiling and the impact of gyrase on relaxing the associated stress (Sevier et al., 2016; Ancona et al., 2019; Klindziuk et al., 2020). Others have focused on the collective behavior of RNAPs (Brackley et al., 2016; Jing et al., 2018; Chatterjee et al., 2021; Geng et al., 2022; Sevier and Hormoz, 2022; Tripathi et al., 2022). In particular, several scenarios have been proposed for the observation of non-trivial long-distance effects associated with transcription. Namely, opposite tendencies for the translocation speed of an RNAP in the presence of other RNAPs have been observed, depending on whether the promoter is active or not, with more rapid, slower respectively, translocation rates (Kim et al., 2019). These phenomena cannot be explained by a simple cancelation of the supercoiling between successive RNAPs (Figure 8). Additional mechanisms have thus been hypothesized. These include (i) the velocity of an RNAP that depends on the net torque that is exerted on it, i.e., the downstream torque minus the upstream torque (Chatterjee et al., 2021; Geng et al., 2022; Sevier and Hormoz, 2022; Tripathi et al., 2022), (ii) a supercoiling stress that increases with the number of bound RNAPs (Chatterjee et al., 2021), (iii) a DNA-bound transcription factor, or a small DNA loop, acting as a topological barrier (Chatterjee et al., 2021), and (iv) a slow diffusion of the linking number (Brackley et al., 2016; Geng et al., 2022).

Hypothesis (i) deserves experimental testing since single-molecule experiments have thus far examined the impact of downstream and upstream torques on elongating RNAPs *separately* (Ma et al., 2013; Ma and Wang, 2014). It also remains to be determined whether this hypothesis is consistent with an elongating RNAP's ability to act as a topological barrier. Lastly, it should be noted that quantitative modeling of transcription by separately considering downstream and upstream stalling torques is feasible (Boulas et al., 2023). Hypothesis (ii) echoes the observation of RNAPs that cluster when the most downstream one stalls (Fujita et al., 2016), which should indeed exert a higher torsional friction. Hypothesis (iii) could be tested experimentally. Nevertheless, both experiments (Leng et al., 2011) and polymer simulations (Joyeux and Junier, 2020) already suggest that, for DNA-bound proteins to generate a topological domain, they must embed the domain inside a loop at the very least. Finally, hypothesis (iv) was made by considering the relaxation speed of the linking number as given by the diffusion speed of plectonemes. However, both single-molecule experiments (Crut et al., 2007; van Loenhout et al., 2012) and polymer simulations (Matek et al., 2015; Joyeux and Junier, 2020; Fosado et al., 2021; Wan and Yu, 2022) have shown that the former, which is responsible for the formation of plectonemes, is much higher than the latter. In other words, supercoiling establishment during transcription can be regarded as a quasi-static process (Wan and Yu, 2022).

Recently, two physical implementations of the TTL model have, for the first time, *separately* considered the actions of Topo I and DNA gyrase (Geng et al., 2022; Boulas et al., 2023) (Figure 8). In particular, the model proposed in Boulas et al. (2023) has a minimal number of parameters and, coupled with an experimental realization of the TTL model in *E. coli*, has provided novel, quantitative insights into the operating mode of topoisomerases. Specifically, it predicts that Topo I and DNA gyrase systematically accompany gene transcription by respectively removing negative and positive turns at rates of approximately one to two (negative) supercoils per second and at least two (positive) supercoils per second. These rates are consistent with *in vitro* activities reported for both Topo I (Terekhova et al., 2012) and DNA gyrase (Ashley et al., 2017). Moreover, the model predicts that the positive linking numbers introduced by Topo I have antagonistic effects on the different stages of transcription. On the one hand, they allow the release of negative torque upstream of the RNAP so that it can properly translocate (Ma et al., 2013; Ma and Wang, 2014). On the other hand, they hinder the opening of the double helix, thereby tending to repress the formation of the so-called open complex (Murakami and Darst, 2003) at the initiation stage.

### 5.3. Open problems and modeling perspectives

#### 5.3.1. Cooperative effects between genes

The global nature of the conservation of the linking number (Section 2) and the quick relaxation of twist and writhe compared to the speed of supercoil generation (Section 4.1) suggest that there is a long-range coupling of supercoiling-induced mechanical stresses that extends to topological barriers. Accordingly, changes in supercoiling around highly transcribing genes can extend up to tens of kb (Visser et al., 2022). Multiple experimental studies have, *de facto*, demonstrated that supercoiling-induced coupling affects the transcription of neighboring genes (Lilley et al., 1996; Opel and Hatfield, 2001), with an impact observed at distances of several kb (Hanafi and Bossi, 2002; Moulin et al., 2004). Physical models have been developed in order to better understand these effects (Meyer and Beslon, 2014; Yeung et al., 2017; Geng et al., 2022; Johnstone and Galloway, 2022; Sevier and Hormoz, 2022) and to understand the impact of this coupling on the organization of genomes (Sobetzko, 2016; Geng et al., 2022) and their possible evolution (Grohens et al., 2022). So far, models have not included effects from topoisomerases, except in a very recent work (Geng et al., 2022). Yet, the necessity to include them to understand the coupling between neighbor genes was stressed (already) 30 years ago in an analysis of the non-trivial transcriptional properties of the leucine biosynthetic operon in *Salmonella* Typhimurium (Lilley and Higgins, 1991). The latter has become a prototypical system of the supercoiling-based coupling of the transcription of divergent genes (Lilley et al., 1996; Rhee et al., 1999; Opel and Hatfield, 2001; Hanafi and Bossi, 2002).

#### 5.3.2. Transcriptional bursting and its time scale

The transcription of many genes in bacteria (and eukaryotes Coulon et al., 2013) has been shown to be bursty (Golding et al., 2005): it is governed by a non-Poissonian process of transcript production involving at least two distinct characteristic times. Namely, single-cell experiments have revealed that the dynamics of expression alternate slowly between active and inactive phases of transcription, with a characteristic time on the order of ten minutes (Golding et al., 2005; So et al., 2011). This characteristic time is much larger than those associated with the mechanisms of transcription during the active phase, whether it be the time required to transcribe the entire gene (~1 minute) or the time between two supercoil removals by the topoisomerases (a few seconds) (Boulas et al., 2023). Importantly, this slow modulation of transcription depends on the activity of DNA gyrase, and the characteristic time for this modulation decreases as the concentration of DNA gyrase increases (Chong et al., 2014). The commonly accepted rationale is the following. RNAPs stall when the positive downstream supercoiling becomes too intense (Ma et al., 2013; Ma and Wang, 2014), that is, when the supercoiling density is on the order of +0.06 (see Section 5.1). In the absence of DNA gyrase, transcription is therefore hindered up to the point where a DNA gyrase binds downstream and relaxes the positive supercoils. These observations raise important questions about the dynamics of the expression of gyrase itself. In particular, is gyrase transcription bursty? Also, measurements in *E. coli* have led to the conclusion that only about 300 gyrases might be bound at each instant along the genome (Stracy et al., 2019), that is, one gyrase every ~15 kb. While this is consistent with DNA gyrase being a limiting factor for transcription, it is not clear why the cell would actually hinder transcription elongation.

#### 5.3.3. The impact of DNA folding

So far, physical implementations of the TTL model have discarded geometrical effects associated with both the one-dimensional sequence-dependent distribution of torsional stress and the three-dimensional folding of DNA, which may impact the binding properties of RNAPs and topoisomerases. Experimentally, the effect of local DNA folding on transcription is actually not known, except in the specific case of small DNA loops involving transcription factors (Cournac and Plumbridge, 2013). Interestingly, Wang suspected that for large values of supercoiling density, folding effects would limit the accessibility of RNAP to DNA (Wang, 1974). His reasoning came from the comparison of two phenomena, whose behaviors as a function of the supercoiling density were similar. Namely, on the one hand, he observed that the transcriptional activity of an RNAP, and more specifically of the core enzyme (i.e., without the ability of the RNAP to recognize specific promoters), is a non-monotonic function of supercoiling density with a maximum at values between −0.05 and −0.04. On the other hand, he observed a change in the sedimentation properties of plasmids in migration gels around −0.035 that he interpreted as a “higher twisting of one double helix around the other” (Wang, 1974). Years later, equilibrium studies of polymer physics models of 10 kb long supercoiled molecules confirmed this conformational effect (Krajina and Spakowitz, 2016): when the supercoiling density decreases below ~ −0.03, branches become longer and tighter, which could indeed hinder accessibility to DNA. We note, here, that this structural effect could actually contribute to the systematic non-monotonic behavior of gene expression level as a function of supercoiling density observed for different promoters *in vitro* (Pineau et al., 2022), although the “maximal” supercoiling values differ substantially between promoters (Pineau et al., 2022). In all cases, models of transcription regulation involving the explicit multi-scale structuring properties of DNA remain to be developed.

## 6. Supercoiling constraints and DNA replication

The topological problems behind and ahead of the advancing replication complex, also known as the replisome, are of a different nature. Behind, they involve the intermingling of two molecules: the replicated DNAs. Ahead, they involve a single molecule: the unreplicated DNA. Let us first recall, then, that the DNA polymerase of mesophilic bacteria duplicates DNA at a rate of about 1000 bp per second. Composed of a large number of proteins and, hence, expected to be constrained by a high torsional friction with the surrounding biomolecules of the cytoplasm, the replisome is unlikely to rotate as quick as it introduces supercoils in DNA. Supposing no rotation at all, the replisome would thus introduce ahead on the order of 100 positive supercoils per second. Considering the presence of topological barriers located at a distance on the order of 10 kb (Section 5), the replisome would thus make the DNA supercoiling density ahead vary by an amount of 0.1 every second—see below for the discussion of a rotating replisome. Since DNA replication is directly linked to the ability of bacteria to multiply, it is therefore not surprising that replisome's advancing is accompanied by a high activity of topoisomerases (Khodursky et al., 2000; McKie et al., 2021), and more specifically ahead by DNA gyrases. In this regard, high-speed single-molecule fluorescence imaging has revealed the presence in *E. coli* of clusters containing an average of 12 gyrases (ranging from 2 to ~100) and concomitant with the onset of replication (Stracy et al., 2019). Also, the DNA gyrase of *Bacillus subtilis* has been shown to relax up to 100 supercoils per second in single-molecule experiments (Ashley et al., 2017). In any case, the effective rate of positive supercoils removal *in vivo* remains unknown. We also remind that the removal of positive supercoiling by DNA gyrases is ATP-dependent with an enzymatic cycle involving the hydrolysis of two ATP molecules to remove two supercoils (Wang, 1998).

**Figure 9:**
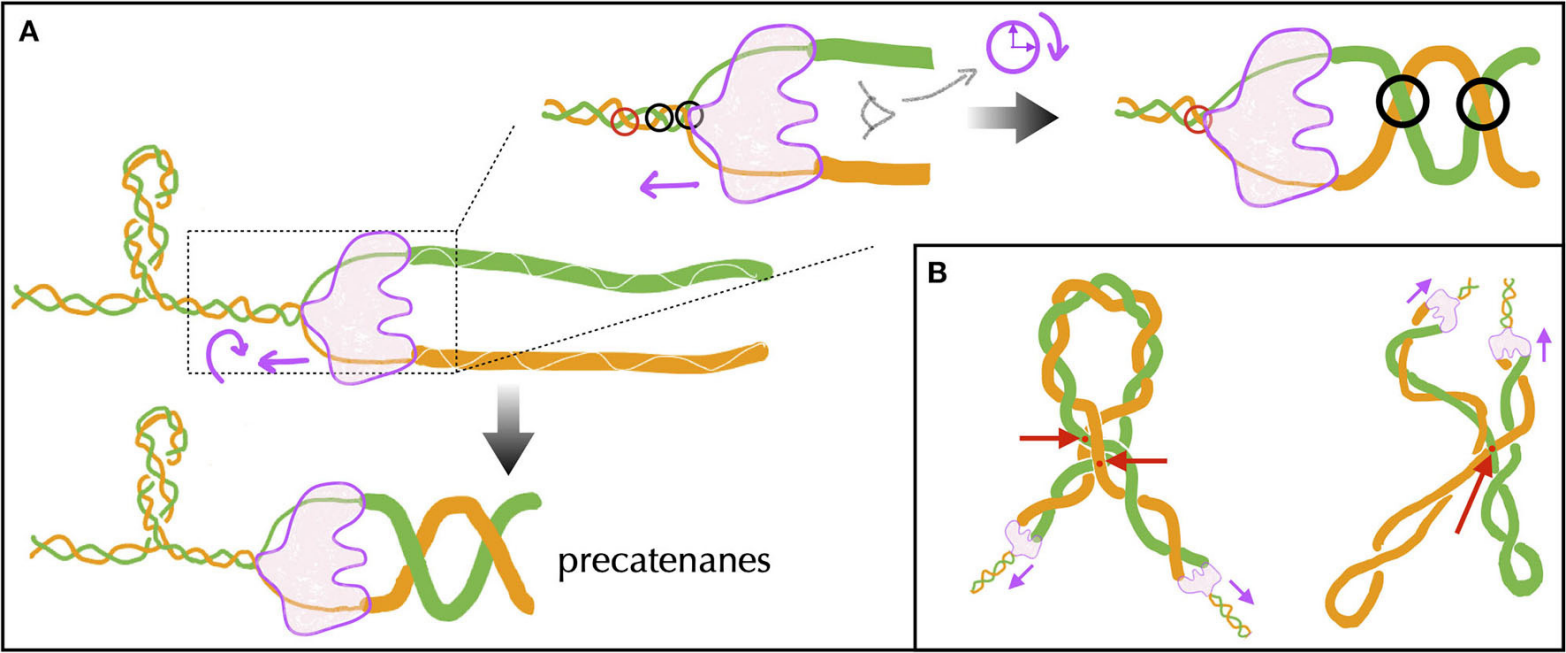
**(A)** Schematic representation of the formation of precatenanes during DNA replication. The replisome, indicated in light purple, moves along the unreplicated DNA double helix with the Watson and Crick strands shown in green and orange, respectively. Behind the replisome, these strands give rise to two replicated molecules indicated by the thick green and orange lines, respectively. Upper panels: during the unwinding of the unreplicated DNA double helix, if the replisome rotates, it transfers the inter-strand crossings (black circles on the left) to the replicated DNA (circles on the right), which then form a superhelix (precatenane) with the same chirality. The red circle indicates a crossing that has not yet been unwound by the replisome. Lower panel: the net result of this operation is the formation of precatenanes. **(B)** Possible conformations of precatenanes leading to left-handed crossings (red points, indicated by the arrows). On the left, the precatenanes buckle to form a plectonemic structure generating two left-handed crossings (adapted from Charvin et al., 2003). On the right, the intrinsic negative supercoiling of each replicated DNA leads to a left-handed crossing (adapted from Rawdon et al., 2016).

Behind the replisome, unwinding of the two DNA strands during replication does not generate mechanical stress that would destabilize the system, as it does in transcription. The two resulting single-stranded DNA molecules are instead managed simultaneously by dedicated enzymes associated with the replication complex to build new double helices (Reyes-Lamothe et al., 2012). However, super-structuring between replicated DNA is known to occur behind the replisome (Peter et al., 1998). To understand this phenomenon, it must be realized that although the replication complex is large, it can rotate in principle, especially because of the large torques generated ahead. From a topological viewpoint, the two replicated DNA molecules extend the Watson and Crick strands of the unreplicated DNA (Figure 9A). The situation is thus identical to the generation of twin supercoils described in Figure 2, with the possibility of rotation of the unwinding machine. According to that figure, the replisome rotates in the clockwise sense, and the replicated DNA forms a right-handed superhelix (Figure 9A), known *in vivo* as precatenanes and in single-molecule experiments as braids. Importantly, precatenanes impede replicated chromosomes from diffusing away from each other. As a consequence, precatenane release is necessary for replicated chromosomes to properly segregate. Multiple lines of evidence over the last 25 years have revealed that this is primarily performed by the topoisomerase Topo IV (Zechiedrich et al., 1997; Charvin et al., 2003; Stone et al., 2003; Wang et al., 2008; Lesterlin et al., 2012), with additional specific contributions from Topo III (McKie et al., 2021).

### 6.1. Polymer models and the precatenane problem

While precatenanes are right-handed, single-molecule experiments have shown that Topo IV decatenates left-handed braided structures much more efficiently (Crisona et al., 2000; Charvin et al., 2003; Stone et al., 2003), raising the question of how Topo IV would remove precatenanes *in vivo*. Three non-exclusive scenarios have been proposed in the context of a polymer physics description of precatenanes. First, equilibrium statistical mechanics analysis of braided molecules have shown that precatenanes, just as DNA, also buckle to form left-handed plectonemes (of precatenanes) when the density of precatenanes is sufficiently high. More precisely, defining the density of precatenanes as the ratio between the number of crossing of the two molecules, on the one hand, and the number of double helices along a single molecule, on the other hand, Marko predicted buckling to occur at a value around 0.045 (Marko, 1997). This has been confirmed by polymer simulations of braided molecules that are stretched by pN-range forces relevant to *in vivo* conditions (Stone et al., 2003; Charvin et al., 2005; Forte et al., 2019). The decatenation of a right-handed precatenane could therefore occur inside a left-handed plectoneme of precatenanes (Figure 9B). Second, for a number of precatenanes much below their buckling regime, single-molecule experiments revealed that the chiral asymmetry in Topo IV activity resulted from a difference in the processivity of the enzymes with respect to the chirality of the braid, with a high (low) processivity for left-handed (right-handed) precatenanes. Topo IV could thus remove right-handed precatenanes, similar to left-handed ones, but at a slower rate. Third, polymer simulations of catenated DNA molecules at equilibrium revealed specific left-handed crossing between the two catenanes when the molecules are negatively supercoiled (Rawdon et al., 2016) (Figure 9B). Accordingly, the decatenation of sister chromatids by Topo IV could be enhanced by negative supercoiling.

In all cases, a puzzling question remains: why would nature select an inefficient Topo IV decatenation activity? A common response is that Topo IV *should not affect the average level of supercoiling*, with the idea that there exists some optimal value of average supercoiling (Menzel and Gellert, 1983). Thus, Topo IV should not intervene in the resolution of right-handed plectonemes generated upstream of RNAPs. However, this answer fails to explain why Topo IV and DNA gyrase have overlapping activities (Hirsch and Klostermeier, 2021; McKie et al., 2021). Moreover, these two enzymes mainly differ at their C-terminal domain only (Hirsch and Klostermeier, 2021), making their inter-conversion a rather easy process from an evolutionary perspective. Instead, we surmise that the inefficiency of Topo IV to remove right-handed plectonemes *allows not to interfere with the dynamics of the transcription initiation stage*. Namely, recent quantitative modeling of transcription (see Section 5 for more details) has demonstrated that transcription initiation is highly sensitive to the action of Topo I. Topo I indeed appears to act both as an elongation facilitator and an initiation inhibitor (Boulas et al., 2023). The reason is that the removal of a single negative supercoil can lead to significant variations in supercoiling at the gene promoter, potentially interfering with the formation of the associated open complex. In this context, an efficient activity of Topo IV on the right-handed plectonemes formed by negative supercoiling could significantly disrupt the delicate balance of Topo I's activity.

### 6.2. The cohesion-segregation problem: insights from polymer models

If not resolved, precatenanes would strongly affect the proper segregation of chromosomal loci. Actually, replicated loci are known to remain close-by in space for at least a few minutes after the passage of the replication machinery. Considering a replication speed of 1000bp per second, this so-called cohesion stage between chromatids thus concerns a post-replicative region that spread over a few hundreds kb. Details of this phenomenon depend on multiple factors, including the type of bacteria, their growth conditions but also the timing along the cell cycle (Possoz et al., 2012; Reyes-Lamothe et al., 2012; Wang et al., 2013; Kleckner et al., 2014; Badrinarayanan et al., 2015). In all cases, specific systems such as an increased activity of Topo IV (El Sayyed et al., 2016) or the action of molecular motors pulling on the replicated DNAs (Bigot et al., 2007) are expected to participate in the resolution of topological problems at the end of replication, when the density of precatenanes is a priori the highest or when only catenanes remain, i.e., when replication is finished. Nevertheless, several *fundamental* aspects of cohesion remain to be understood. For instance, are chromatid cohesion and precatenane formation a unique process, or can chromatids be cohesive without being topologically intermingled? Also, what are the expected respective trajectories of replicated loci once precatenanes are removed? Do they spontaneously segregate? In which directions?

In the early 2000s, the possibility of spontaneous, thermodynamically favorable segregation of intermingled sister chromatids due to the plectonemic structure of each chromatid was proposed (Postow et al., 2001). This was inspired by polymer physics modeling work showing that the probability of catenation between circular DNA and linear cyclizing DNA decreases exponentially with the supercoiling density of circular DNA (Rybenkov et al., 1997)—as a consequence of a volume exclusion from the DNA compacted by the supercoiling and of the reduction of the possibilities to insert the linear DNA into the circular DNA. However, the formation of replication precatenanes is qualitatively different from this problem. A few years later, similar ideas were investigated in the context of the equilibrium statistical mechanics of catenated DNA molecules that are individually supercoiled, asking in particular the question of the amount of energy to provide to add/remove a supercoil to one chromatid vs. add/remove a hypercoil from the pair of concatenated sister chromatids (Martinez-Robles et al., 2009). Two observations were discussed in particular: (i) intra-molecule negative supercoiling under the form of plectonemes make the addition of catenanes more difficult, which may hinder the production of precatenanes; (ii) segregation of the two molecules is favored by plectonemes, very likely as the result of volume-exclusion effects. Knowing that the diffusion of DNA supercoiling stresses is very fast compared to, for example, transcription rates (Ivenso and Lillian, 2016; Joyeux and Junier, 2020; Fosado et al., 2021) (see Section 4 for details), the time scale associated with the structuring of freshly replicated DNA into plectonemes would therefore be dominated by the transcription reinitiation time (i.e., the slowest time scale).

### 6.3. Challenges ahead: out-of-equilibrium models involving long molecules

As discussed above, the mechanisms adopted by Topo IV and, hence, its efficiency to decatenate replicated DNA *in vivo* remain unknown. In particular, the spatial conformations of the precatenanes remain unknown, with at least two types of conformations that could occur (Figure 9B). Moreover, as far as segregation is concerned, it remains to be demonstrated that both volume-exclusion effects and entropic forces similar to those invoked to explain large-scale segregation of chromosomes (Jun and Mulder, 2006; Jun and Wright, 2010) are sufficient to explain the rapid segregation of replicated chromosomes throughout the cell cycle, or whether implication of active-like segregation systems such as the ParABS system (Bouet et al., 2014) is required.

Altogether, these remarks suggest that novel theoretical studies must be performed in order to better understand the disentangling and segregation of freshly replicated chromosomes. In this regard, let us mention a minimal model that has been recently analyzed in the absence of volume exclusion effects (Sevier, 2020). It is composed of three distinct molecules (unreplicated DNA and the two copies of replicated DNA), of a converter that transforms unreplicated DNA double helices into precatenanes as well as the respective actions of DNA gyrase and Topo IV ahead and behind the converter. The objective of this work was to identify very general properties associated with the fundamental constrains on how replisomes and their associated topoisomerases process DNA. The system was analyzed in the simplifying context of a replisome that freely rotates such that the upstream and downstream torques acting on each side of it are equal. Two important results are then worth mentioning. First, in the absence of topoisomerases, it was found that the unreplicated DNA fully collapse into plectonemes before the precatenanes buckle. Second, to avoid this plectonemic collapse, which would trap the replisome, topoisomerases (i.e., DNA gyrase) must remove at least ~ 1 positive supercoil per second.

To further progress in the problem of the disentanglement and the segregation of replicated DNA molecules, it will be necessary to include the explicit structure of DNA, without which the phenomena of volume exclusion are difficult to quantify. The cost to be paid is the absence of analytical solutions and the need to resort to simulations in order to study the far from equilibrium properties of the system. The numerical challenge is significant because the scales involved in the cohesion of sister chromatids (a few hundreds kilo base pairs El Sayyed et al., 2016) are at least one order of magnitude greater than the typical lengths of molecules studied in Brownian dynamics (a few tens kb at most) and two orders of magnitudes greater than the lengths used in the most recent studies of precatenane-like braiding phenomena (Forte et al., 2019). Methods like those used in the dynamics of rigid body (Carrivain et al., 2014) thus need to be contemplated in order to improve the efficiency of the simulations.

These approaches could then give useful information in combination with data about contact frequencies between chromosomal loci, be it those allowing to differentiate sister chromatids as in the recently developed Hi-SC2 method (Espinosa et al., 2020) or those resulting from standard Hi-C methods (Lieberman-Aiden et al., 2009; Le and Laub, 2014). Predictions should be tested in the context of topoisomerase mutants, whose effects on contact properties can be precisely quantified (Conin et al., 2022), and the activity of DNA gyrase and Topo IV hopefully be estimated (at least for various rates of precatenane production). These approaches are also expected to provide crucial insights about how Topo IV actually removes precatenanes *in vivo* by quantifying the relative occurrence of the three mechanisms discussed in Section 6.1 (Figure 9B). These models should also make it possible to validate or refute the spontaneous nature of the segregation of freshly disentangled replicated DNA.

Finally, let us mention that just as eukaryotes, bacteria contain condensins whose activity is crucial to the proper organization and segregation of chromosomes (Hirano, 2016; Gruber, 2018). Interestingly, some of the phenomena associated with the segregation of replicated chromosomes are reminiscent of the problem of the organization and segregation of mitotic chromosomes in eukaryotes (Nasmyth, 2001). Namely, Brownian dynamics simulations in the context of molecular motors extruding DNA have clarified the crucial role of condensins for chromatid segregation during prophase. The proposed mechanism relies on an effective repulsion between topologically unlinked loops (Halverson et al., 2014) facilitated in this particular case by the active extrusion of intra-chromatid DNA loops by the condensins (Goloborodko et al., 2016). Transposed to the problem of bacteria, these approaches offer a promising modeling framework for studying the phenomenology associated with condensins, which are known to play a fundamental role in the segregation of chromosomes (Gruber et al., 2014; Wang et al., 2014; Lioy et al., 2020) and to functionally interact with topoisomerases like Topo IV (Hayama and Marians, 2010; Li et al., 2010).

## 7. Supercoiling and nucleoid formation

**Figure 10:**
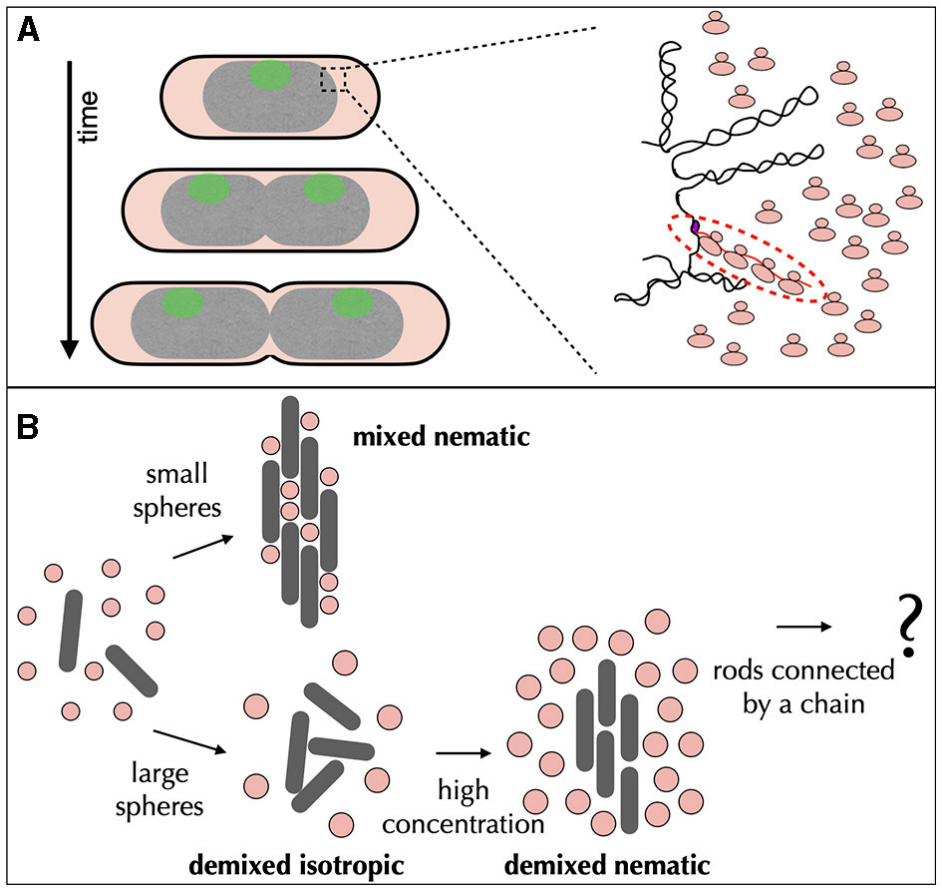
Potential mechanisms involved in the formation of the bacterial nucleoid. **(A)** Left: In rod-shaped bacteria such as *E. coli*, most DNA is localized in the nucleoid (gray area) at the center of the cell, while ribosomes tend to concentrate at the poles (red area). In slowly growing *E. coli* cells, following cell division the origin of replication (in green) is positioned at the center of the cell. During the cell cycle, the replicated origins rapidly segregate toward the quarters of the cell and remain there until cell division occurs. Right: Zooming in on the periphery of the nucleoid, the DNA (plectonemic structures in black) exhibits a tendency to separate from ribosomes (in red). Additionally, polysomes may form when multiple ribosomes simultaneously translate the same messenger RNA (red dashed ellipse). **(B)** In this context, the nucleoid has been proposed to arise from a phase separation process between spherical (ribosomes) and rod-like (plectonemes) structures. Depending on the relative sizes and concentrations of molecular species, at least four scenarios can arise: a well-mixed solution (left) and three distinct phases where rods and spheres undergo demixing (adapted from Urakami and Imai, 2003). The problem for which the rods would be disposed along a polymer chain similar to that expected for the large scale internal structuring of chromosomes (Figure 11) remains open.

Contrary to eukaryotes, bacterial DNA is localized in a membrane-free region of the cell called the nucleoid, which was first highlighted in the 1940s—see Robinow and Kellenberger (1994) for an historical review. Recent live imaging techniques have confirmed this phenomenon, revealing more particularly the exclusion of most ribosomes from the nucleoid so that they localize at the poles of the cells (when these are cylindrical as in many bacteria, Figure 10A)—see Chai et al. (2014) and references therein. In *E. coli*, live fluorescence imaging indicates that, independently of the time point along the cell cycle, the nucleoid occupies approximately half the main axis of the cell and the majority of the cell section, leaving only a thin layer close to the cell wall (Junier et al., 2014; Wu et al., 2019b). Super-resolution techniques have reported smaller and more structured regions (Spahn et al., 2014), in accord with large internal rearrangements occurring at short time scales (i.e., below 1 min) (Wu et al., 2019a). A puzzling aspect of nucleoids has concerned their specific cellular localization during the cell cycle (Possoz et al., 2012; Reyes-Lamothe et al., 2012; Wang et al., 2013; Kleckner et al., 2014; Badrinarayanan et al., 2015). In *E. coli* for instance, just after cell division the nucleoid is localized at the center of the cell. As replication proceeds, it quickly splits into two (replicated) nucleoids which localize at the quarters of the cell until cell division occurs (Figure 10A).

The physical mechanisms responsible for nucleoid formation have fueled numerous theoretical studies (see Benza et al., 2012; Joyeux, 2015 for not too old reviews), with a recurring question: what is the precise role of DNA supercoiling in this matter? The latter is indeed often mentioned as contributing to DNA compaction. However, more than 30 years ago, Boles et al. noted that “the extended thin form of plectonemically supercoiled DNA offers little compaction for cellular packaging, but promotes interaction between cis-acting sequence elements that may be distant in primary structure” (Boles et al., 1990). So, does supercoiling really participate in genome compaction? More specifically, is it a key factor of nucleoid formation?

### 7.1. Spatial extension of a supercoiled DNA vs. confinement: scaling arguments

First and foremost, let us address the question of the spatial extension of a supercoiled circular DNA molecule under conditions of temperature and salinity equivalent to those *in vivo*, but without the confinement of the cell. In polymer physics, the spatial extension of a chain is quantified by its radius of gyration, i.e., the root-mean-square distance between the center of mass of the chain and each of its monomers. It is then customary to describe the large-scale behaviors of polymer chains by assessing how their radius of gyration varies with their molecular length *L* as the latter becomes large, also known as scaling laws (De Gennes and Gennes, 1979). For example, the radius of gyration of both linear and circular self-avoiding chains has been shown to scale as *L*^0.59^ (de Gennes, 1972; Baumgärtner, 1982). Knowing that a circular chain of 30 kb has a radius of gyration on the order of 325nm (see e.g., Walter et al., 2021), this means that a genome of 5Mb (genomic length typical of many bacteria, including *E. coli*) is predicted to have an equivalent radius of gyration of approximately 325 × (5000/30)^0.59^≃*6.6μm*. For comparison, an *E. coli* cell with a length of *2μm* and a radius of *0.5μm* has a much smaller equivalent gyration radius of ≃*0.67μm*. In particular, the volume of the bacterium is (6.6/0.67)^3^ ≈ 1000 times smaller than the typical volume spanned by its thermally fluctuating, unconstrained circular DNA.

As discussed in Section 4, a supercoiled circular DNA molecule adopts tree-like conformations, which is expected to strongly affect these results. Interestingly, by neglecting the details of this tree, such as the distribution of branch sizes, one can estimate the corresponding scaling law. Namely, scaling arguments (Daoud and Joanny, 1981; Khokhlov and Nechaev, 1985; Gutin et al., 1993; Everaers et al., 2017), analytical approaches (Parisi and Sourlas, 1981) and numerical simulations (Rensburg and Madras, 1992; Cui and Chen, 1996; Rosa and Everaers, 2016b, 2017) have shown that the radius of gyration of self-avoiding trees scales as *L*^0.5^. Knowing that a circular chain of 30 kb has a radius of gyration on the order of 200nm in the plectonemic phase (Walter et al., 2021), the corresponding extension for our 5Mb long bacterial genome is equal to 200 × (5000/30)^0.5^≃ 2.6 μm, in accord with more precise calculation (Cunha et al., 2001). While this is a significant reduction compared to topologically unconstrained circular DNA, the corresponding volume is still 60 times larger than the volume of the cells.

### 7.2. Adding (large) molecular crowders: segregative phase separation

The scaling arguments outlined above suggest that supercoiling alone cannot account for the formation of the nucleoid, as the unconfined resulting tree would occupy a much larger volume than the bacterial cell itself. Furthermore, these arguments do not address the specific issue of the nucleoid's location within the cell, whether it is located at the center or the quarters of the cell. One significant factor missing from these arguments is the physical nature of the cytoplasm and the potential for microcompartmentalization caused by liquid-liquid phase separation (Walter and Brooks, 1995; Hyman et al., 2014). In particular, the nucleoid might form due to depletion interactions (Asakura and Oosawa, 1954, 1958; Lekkerkerker and Tuinier, 2011) between the bacterial DNA and “crowders” contained in the cellular solvent in which it is immersed (Odijk, 1998; Mondal et al., 2011; Joyeux, 2020) (Figure 10A).

Molecular crowders are typically identified with small, ~5nm sized proteins, which are present in the cytoplasm in large concentrations. Their presence affects the mobility of biomolecules, protein folding and stability, and the association of macromolecules with each other (van den Berg et al., 2017) as well as the structure and stability of DNA (Miyoshi and Sugimoto, 2008). However, the formation of the nucleoid might owe more to the presence of larger crowders like ribosomes or polysomes (small polymers of ribosomes connected by the messenger RNA they are sitting on) (Mondal et al., 2011; Joyeux, 2020) .

Quite generally, large structures (like spheres, plates or rods) can be pushed together by smaller molecules, as this reduces the total volume inaccessible to the crowders and hence maximizes their translational entropy and the total disorder in the system. In a nutshell, the compressing forces arise because the osmotic pressure of crowders in open spaces cannot be balanced due to their absence from inaccessible spaces. Depletion interactions are particularly effective for rod-like particles, where nematic ordering can arise for similar reasons (Frenkel, 1987) and mixtures of spheres and rod display a rich phase diagram as a function of their relative size and concentration (Figure 10B) (Adams et al., 1998; Dogic et al., 2000; Urakami and Imai, 2003). Of special interest for this review are the implications of DNA supercoiling and, in particular, the importance of the length and the stiffness of the rod-like plectonemic regions in between branch points.

Crowding-induced segregation of plectonemic DNA into a nucleoid was first invoked in 1998 for physiological concentrations of small proteins (Odijk, 1998). However, the equally predicted nematic ordering of the supercoiled DNA has never been observed. Instead, supercoiled DNA appears to mix with small crowders in *in vitro* experiments (Gupta and van der Maarel, 2017) and even with 15nm crowders in Brownian dynamics simulations (Joyeux, 2020). In 2011, a cell-scale model suggested that plectonemic DNA and polysomes undergo segregative phase separation, resulting in a similar phenomenon to that of the nucleoid in *E. coli*: the plectonemes do not exhibit nematic ordering, and the chromosome tends to be localized in the center of the cell, with the polysomes congregating at the poles and in a thin layer between the chromosome and the cell walls (Mondal et al., 2011). The simulation assumed that the crowders had a diameter of 20 nm, slightly larger than in Joyeux (2020), and modeled the bacterial chromosome as a self-avoiding random tree with braided supercoiled DNA branches, approximately 1 kb (200 nm) in size. Notably, the branches were assumed to be *straight, i.e., very stiff* . In this context, the absence of nematic ordering is consistent with previous findings (Urakami and Imai, 2003) where mixtures of rods and spheres with similar diameters exhibited such a phenomenology for a certain concentration of the spheres (Figure 10B). Interestingly, in a mixture of rods of spheres of different sizes, there also exists a regime where the smallest spheres freely mixed with the rods, while the largest spheres may induce the nematic ordering anticipated in Odijk (1998).

Interestingly, in this model of Mondal et al. (2011), the chromosome avoids the cell wall to preserve the orientational entropy of the stiff plectonemes. Even more remarkably, the model predicted that once activated, through the physical coupling of transcription and translation (Section 5), transcribed genes should migrate to the surface of the nucleoid. This was experimentally demonstrated a few years later using live cell super-resolution imaging (Stracy et al., 2015). An important question nevertheless remains: are straight plectonemes of 200nm in size (as used in the model) biologically relevant, knowing that their persistence length is on the order of 100nm, i.e., that they can actually bend rather easily below 200nm? Should one interpret the good agreement between modeling and experimental observations as indirect evidence for the association of plectonemes into stiffer bundles? If not, how would this affect the observed nucleoid phenomenology? Which additional ingredient would be necessary to add in this case? A physical coupling between part of the chromosome and the polysomes to include active genes?

**Figure 11:**
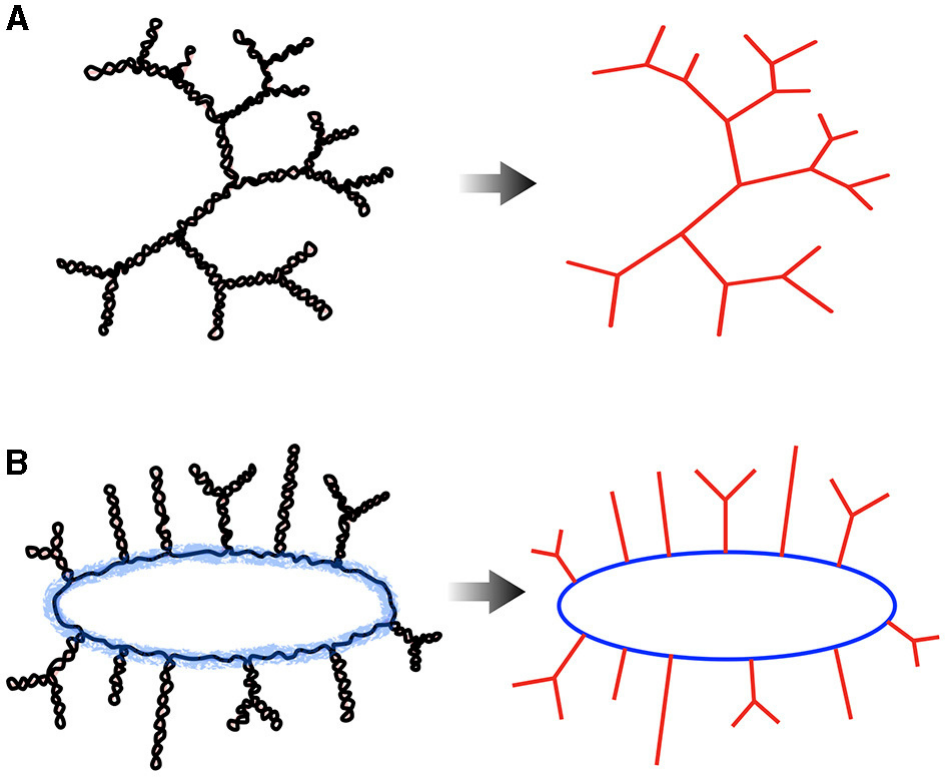
Two types of polymer models including the effects of supercoiling can be contemplated to study the large-scale structure of bacterial chromosomes: **(A)** tree-like models where plectonemes are abstracted by simple linear branches (right panel). **(B)** Bottle brush models where plectonemes are attached along a ring or backbone, indicated in blue. This model is therefore composed of two *a priori* independent entities and, hence, is more complex than the tree-like model. At large scales, the details of these entities can nevertheless be discarded (right panel). It should be noted that if the bottle brush structure is relevant *in vivo*, as suggested by chromosome visualization data in *E. coli* (Wu et al., 2019a), the mechanisms of its formation remain an open question.

Finally, recent visualization of the nucleoid in single non-dividing cells with a growing membrane have shown that a single nucleoid diffuses slowly compared to its internal dynamics, regardless of the cell length. Additionally, it diffuses slowly enough compared to the rate of cell division that it remains at the center of the cell, even when the cell becomes artificially very long (Wu et al., 2019b). To understand this effect, let us first mention that experiments of *E. coli* chromosome micromanipulation have shown that it behaves *in vivo* like a highly compressed spring, meaning that the pressure exerted by the cytoplasm is much greater than that required to fit the chromosome inside the cell (Pelletier et al., 2012). Thus, in a first approximation, the chromosome can be seen as a double-piston for which the cytoplasm exerts strong pressure on each side (Wu et al., 2019b). As the volumes on each side of this piston contain on average equal amounts of proteins, they exert comparable pressure. Nevertheless, the proteins can pass from one side to the other through e.g., the thin layer between the chromosome and the cell wall. The question then is to know the time scale associated with these fluctuations. An interesting insight comes from the modeling work accompanying the experiments of Wu et al. (2019b). Namely, the authors implemented molecular dynamics simulations of a brushed polymer, i.e., of a polymer composed of a (rather stiff) ring to which loops, which could be plectonemes, are attached (Figure 11). This brushed polymer was then immersed in a medium mimicking a cytoplasm crowded by ribosomes. Their results then support the idea that under these conditions, the chromosome diffuses slowly (Wu et al., 2019b), very likely because of rare exchanges of ribosomes between the two sides of the polymer. Accordingly, in the presence of two nucleoids, they showed that the continuous addition of ribosomes distributed equally on either side of the corresponding polymers led to a cellular arrangement with two nucleoids located at the quarters of cells.

In all cases, the same models explaining the formation of the nucleoid as a result of crowding-induced segregation between DNA and ribosomes/polysomes should be able to account for the absence of segregation observed in a few bacteria (Choi et al., 1996). This absence of segregation could correspond to a mixing phase within the space of relevant parameters, such as ribosome density, size, and rigidity of plectonemes. Additionally, it is important to consider that other mechanisms might also play significant roles in the formation of the nucleoid (Joyeux, 2015), including the bridging effect of certain nucleoid-associated proteins (Dillon and Dorman, 2010) or the dynamic formation of loops by bacterial condensins (Hirano, 2016; Gruber, 2018).

## 8. Scaling up models of supercoiled DNA

### 8.1. On trees and bottle brushes

One way to scale up polymer models of supercoiled DNA is to consider that braided structures such as plectonemes behave like self-avoiding linear polymers with, for example, an equivalent diameter of the order of ~10nm for σ = −0.05 (Boles et al., 1990). It then becomes possible to use a classical linear chain modeling without topological constraints (such as a wormlike chain) to address the problem of large-scale polymer folding. In this case, one must nevertheless ask how these pieces of linear chain are connected together. Two possibilities have particularly caught the attention of researchers: tree structures and bottle brush organizations (Figure 11).

The tree-like structures are observed in vitro without the action of enzymes and proteins acting on DNA (Boles et al., 1990) as well as in polymer simulations (see e.g., Krajina and Spakowitz, 2016; Walter et al., 2021 for molecules above 30kb in length). Tree-like models are therefore good candidates to predict behaviors at large scales, i.e., when the details of the trees, such as the length of their branches, do not have an impact on the studied properties—see Daoud and Joanny (1981); Everaers et al. (2017) and references therein for the physics of trees. A characteristic example is the behavior of the average contact frequency between loci as a function of genomic distance (*s*), generally called the “contact law” and denoted by *P*(*s*) (Mirny, 2011). Specifically, in situations of *high polymer concentrations*, simulations of trees lead to contact laws of the form *P*(*s*)~*s*^−1.1^ (Rosa and Everaers, 2019). Interestingly, this law seems to be compatible with observations in very different bacteria, namely *Caulobacter crescentus* (Le et al., 2013), *E. coli* (Lioy et al., 2018), *Pseudomonas aeruginosa* (Varoquaux et al., 2022), or *Streptomyces* (Lioy et al., 2021b).

Remarkably, *P*(*s*)~*s*^−1.1^ is actually also compatible with large-scale contact properties of chromosomal loci in several eukaryotes such as Human (Lieberman-Aiden et al., 2009). While it is tempting to ascribe this here as well to DNA supercoiling known to occur in eukaryotes (Giaever and Wang, 1988; Corless and Gilbert, 2016, 2017), the commonly invoked explanations of crumpling (Grosberg et al., 1993; Cremer and Cremer, 2001; Rosa and Everaers, 2008; Lieberman-Aiden et al., 2009; Mirny, 2011; Halverson et al., 2014) and active loop extrusion (Goloborodko et al., 2016) also lead to double-folded branching structures (Khokhlov and Nechaev, 1985; Grosberg, 2014; Rosa and Everaers, 2014, 2016a, 2019; Everaers et al., 2017). Note, also, that the high concentration nature of the polymers for bacteria is a consequence of an *in vivo* concentration to be considered that is not that of DNA, which is a few percent, but that of plectonemes bound by multiple proteins. Namely, a rough calculation assuming beads of diameter 30nm (consisting of ~10 nm in diameter and ~20 nm of protein complexes) with 200 bp per bead results in a volumetric fraction of beads of approximately 0.7 for a 5Mb genome folded within a nucleoid with a cross-section of 800nm and a length of *1μm*.

Regarding the organization in bottle brush, it should be mentioned firstly that based on biochemical and biophysical analyses of nucleoids extracted from cells, a rosette structure was predicted 50 years ago for the *E. coli* chromosome (Worcel and Burgi, 1972). In this structure, long plectonemes (of approximately 100kb) emanate from a central core made of proteins and RNA. This structure was later confirmed by electron microscopy observations of nucleoids extracted from cells (Kavenoff and Bowen, 1976). However, *in vivo* evidence for such a rosette structure has remained elusive so far. Interestingly, recent experiments in which DNA replication and cell growth were decoupled led to widened cell geometries inside which a toroidal geometry of the circular chromosome of *E. coli* could be clearly identified (Wu et al., 2019a). This structure is compatible with a circular bottle brush polymer model, which is a polymer model made of a circular backbone along which plectonemes are attached (Figure 11B).

Interestingly, the chromosome of *C. crescentus* has been modeled using such a bottle brush polymer model in order to provide a rationale for the patterns observed in the first bacterial Hi-C data produced 10 years ago (Le et al., 2013). In this model, the plectonemes were stochastic structures whose length was adjusted along with 5 other parameters (such as the stiffness of the plectonemes or their distance along the backbone) to reproduce the Hi-C data. Interestingly, the plectonemes in the obtained model had an average length of 15 kb, which is compatible with the length of topologically independent domains predicted to partition bacterial genomes (see Section 4.2). Furthermore, the introduction of plectoneme-free zones blocking the diffusion of plectonemes allowed for the reproduction of the phenomenology of so-called chromosome interaction domains, or CIDs, inside which interaction between any pair of loci is enhanced compared with external loci located at a similar genomic distance (Le et al., 2013). Finally, some of the large-scale conformations of this model adopted a loose helix conformation, a property that has been reported for the *E. coli* chromosome (Hadizadeh Yazdi et al., 2012; Fisher et al., 2013). This is in contrast to the early data-driven “models” of *C. crescentus* chromosomes presenting a marked helix (Umbarger et al., 2011) but whose origin was not physical, as demonstrated in a more physical version of these models by including the fundamental concept of entropy (Messelink et al., 2021). Note also that several theoretical studies have been carried out on these bottle brushes, highlighting helical structures in a regime where the backbone persistence length is at least of the order of the cell diameter (Chaudhuri and Mulder, 2012; Jung and Ha, 2019). The relevance of this hypothesis for *in vivo* situations remains to be demonstrated. Finally, it is noteworthy that the bottle brush polymer model, which was developed for *C. crescentus* (Le et al., 2013), has recently inspired a data-driven approach aimed at creating a three-dimensional representation of the current knowledge on the structuring of bacterial chromosomes (Hacker et al., 2017).

### 8.2. On-lattice models

The simulation of tree-like models is commonly performed on a lattice. Lattice simulations are preferred due to the ease of managing discrete elementary movements as compared to continuous movements involved in off-lattice approaches. This leads to higher efficiency of lattice simulations. In fact, lattice simulations are particularly suitable when the properties under study occur on a much larger scale than the lattice mesh, i.e., when the properties studied do not depend on the geometric parameters of the lattice. The possibility of performing non-local movements, such as cutting a branch at one point and randomly reintroducing it at another point (Seitz and Klein, 1981; Rensburg and Madras, 1992; Rosa and Everaers, 2016a,b), or the exchange sections of overlapping chains (Karayiannis et al., 2002) or trees are particularly effective on a lattice (Svaneborg et al., 2016; Svaneborg and Everaers, 2023) and allow to reach thermodynamic equilibrium very efficiently. Elastic chain methods on the lattice are also very effective for exploring polymer dynamics in situations where the polymer concentration is very high (Evans and Edwards, 1981; Bar, 1999; van Heukelum and Barkema, 2003; Hugouvieux et al., 2009; Schram et al., 2013). In a nutshell, the principle is based on the ability to redistribute monomers along a given spatial conformation, with several consecutive monomers being able to overlap. This then allows for the exploration of new conformations that would be inaccessible without this prior redistribution. Finally, simulation techniques can be adopted to reproduce realistic dynamic properties of polymers (Ghobadpour et al., 2021), as well as simulate active processes such as the action of condensins (Miermans and Broedersz, 2020).

To our knowledge, no work has reported on the properties of a lattice-based physical model that covers the multiple scales of the bacterial chromosome. However, it is worth mentioning that a computational method has been developed to efficiently construct lattice-based conformations of a bottle brush polymer with a backbone to which plectonemes are attached (Goodsell et al., 2018). The plectoneme modeling used in this study bears resemblance to the double-folding lattice polymer models, where linear chains fold back on themselves to form overlapping double-chain structures. In this regard, we believe that the range of methods developed in this specific area of polymer physics should allow for a precise and quantitative analysis of the physical nature of bacterial chromosomes. Specifically, models should be capable of explaining both contact properties and the spatial positioning of loci identified through fluorescence visualization (Espeli et al., 2008; Hong et al., 2013). Interestingly, it seems that within models, the latter naturally arises from the former when forcing the localization of only a few specific loci, such as those associated with the origin and terminus of replication (Messelink et al., 2021). In this context, an important open question to us is the following: is it possible to distinguish between tree-like and bottle brush-type phenomenologies based solely on contact properties between loci as provided by Hi-C data, knowing that the latter can be generated in principle for any type of bacteria cultivable in the laboratory (Marbouty and Koszul, 2017)?

## 9. Concluding remarks

In this review, we have discussed various models of bacterial supercoiled DNA, which differ in the scales they describe and the types of processes involved. We have specifically distinguished between structuring phenomena that can be described using thermodynamic equilibrium approaches and phenomena that operate far from equilibrium, such as gene transcription or DNA replication.

One fundamental question, which is expected to gain increasing importance, especially within the field of systems biology, is whether it is possible to develop a physically-grounded unified framework that integrates these different modeling perspectives. The challenge lies in developing a multi-scale model of biophysical phenomena, wherein identifying a hierarchy of mechanisms, if it indeed exists, can be extremely difficult. This problem of developing a hierarchy of descriptions is already a challenge in the study of physical matter, particularly in the context of numerical simulation (Steinhauser, 2017). In the case of biological matter, and more specifically in the field of Chromosome Biology, this problem is even more central. A comprehensive understanding of phenomena indeed requires, in principle, considering scales ranging from the base pair level to the cellular organization of chromosomes.

Next, the discussed models often neglects the interactions of DNA with proteins and molecular machines, as well as with all the small molecules and ions that make up the cytoplasm. Although coarse-grained models of (supercoiled) DNA have proven successful in single-molecule experiments, it is therefore reasonable to question how well these models capture the behavior of DNA in a living cell. Even more worrying for a rational approach to the phenomena at play, proteins and molecular machines often have their own specificity that arises from the molecular tinkering induced by natural selection (Jacob, 1977). Many of their properties therefore escape the universality feature of physical phenomena.

The relevance of coarse-grained models nevertheless arises from two realities. First, in many situations, the conditions are equivalent to those of a system with a large number of particles or in the limit of a very large size of the entities involved. In this case, statistical physics approaches become relevant. For example, while the plectonemic structure of supercoiled DNA may be a simplifying average view of the dynamics of DNA interacting with many proteins and molecular machines, this average behavior becomes probably relevant at much larger scales, such as the chromosome, and a tree-like description of the problem should capture a good part of the associated phenomena. Second, evolutionary conserved phenomena are often associated with generic physical properties (Junier, 2014). For instance, the double helix nature of DNA necessarily creates topological problems that require dedicated enzymes to resolve. This has two consequences: first, topoisomerases are ubiquitous in living organisms; and second, generic physical models for handling topological constraints can be considered, regardless of the mechanisms involved. Variations in behavior between bacteria should then reflect the possible range of physiologically relevant parameters. In all cases, proposed physical models should be evaluated not only for their descriptive (i.e., postdictive) capacity but also, and perhaps most importantly, for their predictive power.

## Author contributions

IJ wrote the first draft. IJ, OE, and RE revised the first draft. All authors contributed to the article and approved the submitted version.
