# Supplementary material for: DNA supercoiling in bacteria: state of play and challenges from a viewpoint of physics based modeling
Source: Front Microbiol. 2023 Oct 30;14:1192831. doi: 10.3389/fmicb.2023.1192831 (PMC10642903; doi:10.3389/fmicb.2023.1192831)
Supplement: Supplementary file 1 [file Presentation_1.pdf]

Appendix: A crash introduction to physical modeling of supercoiling-related phenomena

Ivan Junier,[1,*] Elham Ghobadpour,[1,2] Olivier Espeli,[3] and Ralf Everaers[2]

[1] *Univ. Grenoble Alpes, CNRS, UMR 5525, VetAgro Sup, Grenoble INP, TIMC, 38000 Grenoble, France*
[2] *Université de Lyon, École Normale Supérieure (ENS) de Lyon, CNRS, Laboratoire de Physique and Centre Blaise Pascal de l'ENS de Lyon, F-69342 Lyon, France*
[3] *Center for Interdisciplinary Research in Biology (CIRB), Collège de France, CNRS, INSERM, Université PSL, Paris, France*

In this appendix, we aim to provide a concise and accessible introduction to biophysical models of supercoiled DNA. To accomplish this, we first introduce the concept of coarse-graining, which determines the level of detail captured by models. Next, we delve into the principles of Statistical Mechanics, a framework widely used by biophysicists. As an example, we discuss the simplest model for studying the folding properties of supercoiled DNA, known as the rod-like chain model. We then distinguish three main situations, depending whether models are studied or defined i) at thermodynamics equilibrium, ii) out of equilibrium or iii) far from equilibrium. Additionally, we discuss the power of phenomenological approaches, which allow to capture system properties in an approximative, yet often quantitative way. Finally, we discuss the Monte Carlo and Brownian Dynamics methods that are commonly used to simulate the folding of DNA in the context of these models.

All the figures referenced in this appendix are from the main text.

CONTENTS

A1. Coarse-graining level: deciding which details to drop

A2. Statistical mechanics: the example of the rod-like chain model

A3. Equilibrium statistical mechanics and phenomenological approaches

A4. Non-equilibrium models
  Out of equilibrium
  Far from equilibrium

A5. Polymer simulations to investigate the folding of supercoiled DNA
  The Monte Carlo method
  The Brownian dynamics method

References

A1. COARSE-GRAINING LEVEL: DECIDING WHICH DETAILS TO DROP

The first step in constructing a physical model of DNA involves determining the level of approximation, known as the coarse-graining level, which defines the spatial and temporal scales below which structural and mechanistic details are discarded. For instance, studying the effects of DNA supercoiling does not require explicit consideration of the quantum physics of atoms and chemical bonds. The most detailed models actually operate at the resolution of individual nucleotides [1–3]. Their applicability is nevertheless limited to relatively small molecules due to the time-consuming nature of simulations involved. In the review, we discuss coarse-graining approaches above the double helix, typically spanning tens of base pairs or more. These models allow investigation of properties at scales ranging from kilobase pairs to megabase pairs. Importantly, models with resolution above the double helix neglect the specific structure of the double helix itself. As a consequence, they necessitate the inclusion of an effective treatment for conserving the linking number (see below).

* ivan.junier@univ-grenoble-alpes.fr

A2. STATISTICAL MECHANICS: THE EXAMPLE OF THE ROD-LIKE CHAIN MODEL

Once the coarse-graining level is chosen, a model of DNA can be constructed using the principles of Statistical Mechanics. This branch of physics focuses on predicting the macroscopic properties of systems comprised of microscopic entities. The specific internal and interaction properties of these entities determine the parameters of the models. A classic example related to DNA supercoiling phenomena is the rod-like chain model [4, 5]. In this model (Fig. 4A), DNA is represented as a series of articulated rigid segments, where the relative orientation of each segment is constrained by two parameters: the bending and torsional moduli. These parameters quantify the resistance of DNA to bending and torsion, respectively. Typically, the associated energy costs are expressed as quadratic functions of the differences in tangent and, respectively, angular orientations between two adjacent segments (Fig. 4A), with a proportionality constant specified by the moduli. Next, the conservation of the linking number can be implemented "locally" using the "parallel transport" approach [6], which imposes a specific analytical form for the relationship between twist and the relative orientation of contiguous segments – the relative orientation of the Euler frames associated with each segment, to be more precise [7, 8] (Fig. 4A). It is worth noting that the twist has actually often been defined using the very angles characterizing the Euler frame, which do not satisfy the condition of "parallel transport". However, in this case, the linking number only exhibits slight fluctuations around its expected value, making this Euler-based definition a valid practical approximation [9].

A common macroscopic property explored in the rod-like model is the spatial extension of the molecule and how it relates to the supercoiling density. In this regard, it is important to note that this model allows segments to overlap in space. More realistic models can be constructed by incorporating electrostatic repulsions between segments, leading to the self-avoiding rod-like chain model [4]. Additionally, alternative forms of DNA can be considered [8, 10–12]. By utilizing these refined models, macroscopic properties like the fraction of super-structuring or the fraction of denatured DNA monomers can be examined. Finally, if DNA/RNA polymerases and topoisomerases are incorporated into the model, the system becomes more complex, requiring the consideration of additional parameters to fully describe it. These include, for example, the rates at which topoisomerases remove supercoils or the speed at which RNA/DNA polymerases translocate along DNA. Additional macroscopic properties relevant to the functioning of bacteria can then be examined such as the production rate of RNA transcripts.

A3. EQUILIBRIUM STATISTICAL MECHANICS AND PHENOMENOLOGICAL APPROACHES

The simplest scenario for investigating properties in models like the rod-like chain model is when all system changes can be attributed solely to thermal energy exchanges with the solvent (cytoplasm).This assumption establishes the framework of "equilibrium statistical mechanics", which states that the probability of any molecular configuration of the chain is proportional, at long times, to the Boltzmann weights: $\exp[-E/k_B T]$. E represents the energy of the configuration, reflecting bending and torsional costs, k_B is the Boltzmann constant, and T is the cytoplasm's temperature. In certain cases, such as in the regime of low supercoiling where plectonemes of the actual DNA molecule are not distinguishable, it becomes possible to precisely calculate the average and variance of properties like the spatial distance of the DNA chain [5, 13]. For example, in 1998, Bouchiat and Mezard presented a semi-analytical solution (involving the numerical solution of a system of two equations) for the average spatial extension of the continuous version of the rod-like chain model, as a function of supercoiling density and the stretching force acting on it [13]. Their results demonstrated excellent agreement with those obtained from single molecule experiments [5, 13, 14].

In the most general case, deriving exact or nearly exact solutions for equilibrium properties of statistical systems is nevertheless not feasible. Even for the simplest models, calculations indeed become quickly insurmountable, as in the rod-like chain model when super-structuring becomes dominant [5]. As a result, alternative methods need to be considered. In this regard, *bona fide* thermodynamics formalisms, parametrized by the same parameters as those in the underlying statistical system (e.g., supercoiling density), have often proved to be powerful. In particular, approximative solutions can be derived by minimizing the corresponding free energy, which incorporates the interplay between energy and entropy costs that govern the macroscopic behavior of the system. The relevance of these so-called phenomenological approaches lies in the profound connection between statistical mechanics and thermodynamics, where the former provides a microscopic foundation for the latter. The primary challenge then lies in determining a functional form of the free energy that accurately captures the statistical properties of the original system. An illustrative example is the work of Siggia and Marko in 1994, who tackled the issue of super-structuring in a supercoiled DNA chain within the framework of the self-avoiding rod-like chain model. Through the utilization of such a phenomenological approach, they were able to provide an explanation for the higher likelihood of plectonemes (Fig. 5) compared to toroids, although both types of super-structures may possess the same writhe values [15].

A4. NON-EQUILIBRIUM MODELS

Out of equilibrium

While conditions are such that a system is expected to reach thermodynamics equilibrium at long times, the relaxation time required for this equilibrium state to be achieved can be so long that the system may effectively remain out of equilibrium. A prototypical example is the formation of the crumpled (or fractal) globule, a conformation of polymer chains that is predicted to generally occur when the chains are prevented from becoming entangled [16] – a situation expected to be relevant for the functioning of cellular DNA. Numerical simulations of high concentrations of polymer chains have revealed that the crumpled globule is a metastable, out of equilibrium conformation that inevitably arises during the swelling of initially condensed, untangled chains, such as those associated with the mitotic chromosomes of eukaryotes [17, 18] – see section 8 of the main text for the relevance to the problem of large-scale models of supercoiled DNA. So, while the equilibrium likelihood of the crumpled globule is very low, its lifetime may be so large (hundreds years in the case of the human genome [17]) that it becomes more relevant than the equilibrium, highly entangled globule. The term "out of equilibrium" is then used to describe a situation where thermodynamic concepts, such as free energies, are still applicable in capturing the properties of the corresponding systems. This includes systems that are in the process of reaching thermal equilibration, like the crumpled globule, as well as systems in which perturbations from thermodynamic equilibrium are sufficiently small that their properties can be predicted by considering small deviations from equilibrium statistical mechanics.

Far from equilibrium

In many situations, such as when gyrase utilizes ATP to relax positive supercoils, an additional energy source is required apart from thermal energy. Furthermore, during DNA replication or gene transcription, an influx of matter (nucleotides) is necessary to produce new entities. In these scenarios, describing the system thermodynamically, even in an approximate manner, is often impossible. The associated systems and models are then referred to as being "far from equilibrium". Oscillatory systems, which are prevalent in cellular processes, are a prototypical example that cannot be encompassed within an equilibrium framework since, by definition, they do not satisfy temporal invariance of equilibrium properties.

Interestingly, while gene transcription and DNA replication involve far from equilibrium situations, equilibrium statistical mechanics can still be relevant in explaining certain properties. For example, the general sensitivity of gene transcription to DNA supercoiling can be rationalized using equilibrium-like models [19]. This suggests that thermal activation often plays a crucial role in the limiting steps of transcription. A notable example is when the process is primarily limited by the stage where the DNA, bound by the RNAP, must denature to form the open complex. In this case, the rate of transcription is largely determined by the thermodynamic stability of the DNA duplex at the promoter [20, 21].

A5. POLYMER SIMULATIONS TO INVESTIGATE THE FOLDING OF SUPERCOILED DNA

Due to the inherent complexity of solving even the simplest model, such as the rod-like chain, numerical simulations of polymer chains are frequently necessary to investigate the folding properties of DNA, whether it is supercoiled or not. This section explains the principles of two commonly used methods for conducting such simulations.

The Monte Carlo method

One of the most commonly used approaches to simulate an equilibrium situation is the Monte Carlo Markov chain method, often referred to as the Monte Carlo method [22]. The algorithm is relatively straightforward. It involves iterating through a process in which a random entity from the system is selected, and one of its properties is updated with a certain probability. In the context of thermodynamic equilibrium, this probability depends on the change in energy associated with the attempted update and follows a rule known as the "detailed balance condition". This condition ensures that, given a sufficiently large number of iterations, the system will reach thermodynamic equilibrium.

For a circular rod-like chain, a typical trial involves randomly selecting two articulation points and rotating the segments located between them by a randomly chosen angle (Fig. 4B). This motion, known as a crankshaft motion,

alters the bending and torsion energies between the segments at the articulation points and is accepted, or not, following the detailed balance condition. By forbidding rotations that cause segments to intersect in space, the simulated model becomes a self-avoiding rod-like chain.

The Brownian dynamics method

Monte Carlo methods can be efficient in rapidly reaching thermal equilibrium. However, the dynamics they simulate is not realistic, particularly when global moves occur, such as during the rotation of a large number of segments. Therefore, caution must be taken when applying these methods to capture the dynamical properties of real molecules. Similarly, without considering specific types of moves/updates [23], Monte Carlo "dynamics" may not be compatible with certain types of motions that do occur in real molecules, such as the slithering of plectonemes. To solve these problems and enable a more realistic simulation of the dynamics of DNA, Brownian dynamics simulations [24, 25], which relax the constrain of the rigid segment, are often used. To this end, the DNA chain is described in terms of beads [26, 27] (Fig. 4C) and its motion is simulated by considering the equations of movement for the beads. Namely, Brownian dynamics simulations assume that DNA beads experience significant frictional forces in the cytoplasm so that their inertia can be neglected. The equations of motion are then numerally solved by updating the positional and rotational degrees considering two main types of forces: (i) those that derive from potential energies, which include the artificial "bond energy" between contiguous beads, the bending and torsional energies of the original rod-like chain model, and the short-distance energies of electrostatic repulsion; and (ii) random forces associated with thermal energy, which is responsible for the translational and rotational diffusion of the beads [26]. Eventually, by introducing a third category of forces known as active forces [28], it becomes possible to simulate far from equilibrium situations, such as the generation of supercoiling due to transcription [29, 30].

In practice, Brownian dynamics methods allow to simulate in a reasonable amount of time, that is, in less than a few months, 20 kb long chains for a total of typically one millisecond [31]. This leads to two important remarks. Firstly, similar to Monte Carlo methods, these methods are limited to chains that are typically two orders of magnitude smaller than the typical length of bacterial genomes (megabase pairs). Alternative modeling approaches are thus required to handle larger systems as discussed in section 8 of the main text. Secondly, these methods are not suitable for simulating processes with characteristic time scales on the order of minutes, such as gene transcription or DNA replication. This explains why gene transcription is currently modelled as one-dimensional stochastic processes along the DNA – see section 5 of the main text for more details.

---

[1] S. A. Harris, Modelling the biomechanical properties of DNA using computer simulation, Philosophical Transactions of the Royal Society A: Mathematical, Physical and Engineering Sciences **364**, 3319 (2006).

[2] T. E. Ouldridge, A. A. Louis, and J. P. K. Doye, Structural, mechanical, and thermodynamic properties of a coarse-grained DNA model, The Journal of Chemical Physics **134**, 085101 (2011).

[3] M. Manghi and N. Destainville, Physics of base-pairing dynamics in DNA, Physics Reports **631**, 1 (2016).

[4] A. V. Vologodskii, S. D. Levene, K. V. Klenin, M. Frank-Kamenetskii, and N. R. Cozzarelli, Conformational and thermodynamic properties of supercoiled DNA, Journal of Molecular Biology **227**, 1224 (1992).

[5] C. Bouchiat and M. Mézard, Elastic rod model of a supercoiled DNA molecule, The European Physical Journal E **2**, 377 (2000).

[6] M. Bergou, M. Wardetzky, S. Robinson, B. Audoly, and E. Grinspun, Discrete elastic rods, in *ACM SIGGRAPH 2008 papers* (ACM, Los Angeles California, 2008) pp. 1–12.

[7] P. Carrivain, M. Barbi, and J.-M. Victor, In Silico Single-Molecule Manipulation of DNA with Rigid Body Dynamics, PLoS Computational Biology **10**, e1003456 (2014).

[8] T. Lepage and I. Junier, A polymer model of bacterial supercoiled DNA including structural transitions of the double helix, Physica A: Statistical Mechanics and its Applications **527**, 121196 (2019).

[9] A. V. Vologodskii and N. R. Cozzarelli, Conformational and Thermodynamic Properties of Supercoiled DNA, Annual Review of Biophysics and Biomolecular Structure **23**, 609 (1994).

[10] M. Manghi, J. Palmeri, and N. Destainville, Coupling between denaturation and chain conformations in DNA: stretching, bending, torsion and finite size effects, Journal of Physics: Condensed Matter **21**, 034104 (2009).

[11] A. K. Efremov, R. S. Winardhi, and J. Yan, Transfer-matrix calculations of DNA polymer micromechanics under tension and torque constraints, Physical Review E **94**, 032404 (2016).

[12] T. Lepage and I. Junier, Modeling Bacterial DNA: Simulation of Self-Avoiding Supercoiled Worm-Like Chains Including Structural Transitions of the Helix, in *The Bacterial Nucleoid*, Vol. 1624, edited by O. Espéli (Springer New York, New York, NY, 2017) pp. 323–337, series Title: Methods in Molecular Biology.

[13] C. Bouchiat and M. Mézard, Elasticity Model of a Supercoiled DNA Molecule, Physical Review Letters **80**, 1556 (1998).

[14] T. R. Strick, M.-N. Dessinges, G. Charvin, N. H. Dekker, J.-F. Allemand, D. Bensimon, and V. Croquette, Stretching of macromolecules and proteins, Reports on Progress in Physics **66**, 1 (2003).

[15] J. F. Marko and E. D. Siggia, Fluctuations and Supercoiling of DNA, Science **265**, 506 (1994).

[16] A. Grosberg, Y. Rabin, S. Havlin, and A. Neer, Crumpled globule model of the three-dimensional structure of dna, Europhysics Letters **23**, 373 (1993).

[17] A. Rosa and R. Everaers, Structure and dynamics of interphase chromosomes, PLOS Computational Biology **4**, 1 (2008).

[18] E. Lieberman-Aiden, N. L. van Berkum, L. Williams, M. Imakaev, T. Ragoczy, A. Telling, I. Amit, B. R. Lajoie, P. J. Sabo, M. O. Dorschner, R. Sandstrom, B. Bernstein, M. A. Bender, M. Groudine, A. Gnirke, J. Stamatoyannopoulos, L. A. Mirny, E. S. Lander, and J. Dekker, Comprehensive Mapping of Long-Range Interactions Reveals Folding Principles of the Human Genome, Science **326**, 289 (2009).

[19] M. Pineau, S. Martis B., R. Forquet, J. Baude, C. Villard, L. Grand, F. Popowycz, L. Soulère, F. Hommais, W. Nasser, S. Reverchon, and S. Meyer, What is a supercoiling-sensitive gene? Insights from topoisomerase I inhibition in the Gram-negative bacterium *Dickeya dadantii*, Nucleic Acids Research **50**, 9149 (2022).

[20] R. Forquet, M. Pineau, W. Nasser, S. Reverchon, and S. Meyer, Role of the Discriminator Sequence in the Supercoiling Sensitivity of Bacterial Promoters, mSystems **6**, 10.1128/mSystems.00978-21 (2021).

[21] R. Forquet, W. Nasser, S. Reverchon, and S. Meyer, Quantitative contribution of the spacer length in the supercoiling-sensitivity of bacterial promoters, Nucleic Acids Research **50**, 7287 (2022).

[22] D. Frenkel and B. Smit, *Understanding molecular simulation: from algorithms to applications*, Vol. 1 (Elsevier, 2001).

[23] Z. Liu and H. S. Chan, Efficient chain moves for Monte Carlo simulations of a wormlike DNA model: Excluded volume, supercoils, site juxtapositions, knots, and comparisons with random-flight and lattice models, The Journal of Chemical Physics **128**, 145104 (2008).

[24] S. A. Allison and J. A. McCammon, Multistep brownian dynamics: Application to short wormlike chains, Biopolymers **23**, 363 (1984).

[25] G. Chirico and J. Langowski, Calculating hydrodynamic properties of DNA through a second-order Brownian dynamics algorithm, Macromolecules **25**, 769 (1992).

[26] G. Chirico and J. Langowski, Kinetics of DNA supercoiling studied by Brownian dynamics simulation, Biopolymers **34**, 415 (1994).

[27] H. Jian, A. V. Vologodskii, and T. Schlick, A Combined Wormlike-Chain and Bead Model for Dynamic Simulations of Long Linear DNA, Journal of Computational Physics **136**, 168 (1997).

[28] P. Romanczuk, M. Bär, W. Ebeling, B. Lindner, and L. Schimansky-Geier, Active Brownian particles: From individual to collective stochastic dynamics, The European Physical Journal Special Topics **202**, 1 (2012).

[29] Y. A. G. Fosado, D. Michieletto, C. A. Brackley, and D. Marenduzzo, Nonequilibrium dynamics and action at a distance in transcriptionally driven DNA supercoiling, Proceedings of the National Academy of Sciences **118**, e1905215118 (2021).

[30] M. Joyeux, Models of topological barriers and molecular motors of bacterial DNA, Molecular Simulation **48**, 1688 (2022).

[31] M. Joyeux and I. Junier, Requirements for DNA-Bridging Proteins to Act as Topological Barriers of the Bacterial Genome, Biophysical Journal **119**, 1215 (2020).
